# Analysis of the Central Nervous System Transcriptome of the Eastern Rock Lobster *Sagmariasus verreauxi* Reveals Its Putative Neuropeptidome

**DOI:** 10.1371/journal.pone.0097323

**Published:** 2014-05-12

**Authors:** Tomer Ventura, Scott F. Cummins, Quinn Fitzgibbon, Stephen Battaglene, Abigail Elizur

**Affiliations:** 1 Faculty of Science, Health, Education and Engineering, GeneCology Research Centre, University of the Sunshine Coast, Sunshine Coast, Queensland, Australia; 2 Institute for Marine and Antarctic Studies, University of Tasmania, Hobart, Tasmania, Australia; University of Rouen, France

## Abstract

Neuropeptides have been discovered in many arthropod species including crustaceans. The nature of their biological function is well studied and varies from behavior modulation to physiological regulation of complex biochemical processes such as metabolism, molt and reproduction. Due to their key role in these fundamental processes, neuropeptides are often targeted for modulating these processes to align with market demands in commercially important species. We generated a comprehensive transcriptome of the eyestalk and brain of one of the few commercially important spiny lobster species in the southern Hemisphere, the Eastern rock lobster *Sagmariasus verreauxi* and mined it for novel neuropeptide and protein hormone-encoding transcripts. We then characterized the predicted mature hormones to verify their validity based on conserved motifs and features known from previously reported hormones. Overall, 37 transcripts which are predicted to encode mature full-length/partial peptides/proteins were identified, representing 21 peptide/protein families/subfamilies. All transcripts had high similarity to hormones that were previously characterized in other decapod crustacean species or, where absent in crustaceans, in other arthropod species. These included, in addition to other proteins previously described in crustaceans, prohormone-3 and prohormone-4 which were previously identified only in insects. A homolog of the crustacean female sex hormone (CFSH), recently found to be female-specific in brachyuran crabs was found to have the same levels of expression in both male and female eyestalks, suggesting that the CFSH female specificity is not conserved throughout decapod crustaceans. Digital gene expression showed that 24 out of the 37 transcripts presented in this study have significant changes in expression between eyestalk and brain. In some cases a trend of difference between males and females could be seen. Taken together, this study provides a comprehensive neuropeptidome of a commercially important crustacean species with novel peptides and protein hormones identified for the first time in decapods.

## Introduction

The Eastern rock lobster *Sagmariasus verreauxi* is one of a few closely related species which constitute the spiny lobster fishery industry in the Southern Hemisphere [1]. Identifying the molecular components which govern fundamental processes in this species might thus prove useful in further enhancing the aquaculture industry of this taxonomic group. Neuropeptides and protein hormones have long been suggested as targets for crustacean aquaculture enhancement [2], [3]. They govern a wide array of physiological and behavioral processes and have been studied extensively in crustaceans [4]. Neuropeptides are translated as larger precursors (usually known as prepro-peptides) which include a signal peptide at their N-terminus. The signal peptide directs the prepro-peptide translation into the rough endoplasmic reticulum, where the signal peptide is being cleaved off, leaving the pro-peptide which is then further processed prior to the secretion of the mature peptide [4].

The list of putative neuropeptide sequences from different crustacean species has considerably increased over the past few years with the employment of bioinformatic mining in publicly available databases [5], *de novo* transcriptome assemblies [6]–[9] and mass spectrometry [10]–[13]. With the expansion of the crustacean neurohormone database, identification of the conserved features of the mature neurohormones further enables mining of novel neurohormones through *de novo* transcritomes of crustacean species where neurohormones were not previously identified. Comparisons with other arthropod species where neuropeptidomes have been characterized [14]–[21] enable insights into species' life history as in the case of the parasitic wasp *Nasiona vitripennis* [14] and the social honeybee *Apis mellifera* [15] and evolution, as in the case of the fruit fly *Drosophila sp.* [19] and the silk moth *Bombyx mori* [20].

With the recent rapid advancement in transcriptome sequencing capabilities, it becomes increasingly affordable to establish comprehensive transcriptomes of non-model organisms. We collected RNA from several key tissues that are known to be the primary sites of neuropeptide production and secretion in crustaceans and generated a comprehensive transcriptome of *S. verreauxi*. These tissues included the eyestalk, where the X-organ-sinus gland (XOSG) neuroendocrine complex resides, the thoracic ganglia and brain. From the transcriptomic data obtained, we compiled a list of the putative neuropeptides and protein hormones and characterized them via comparisons to previously reported neuropeptides to predict the processing of prepro-peptides into mature neuropeptides. The conserved motifs were identified and highlighted, providing a database that might prove useful for further identification of neuropeptides in closely related species.

## Results

### Allatostatins

**Figure 1:**
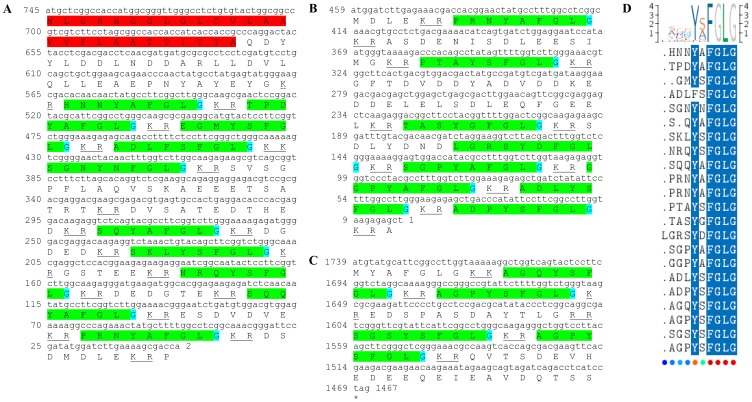
Type A allatostatin precursors predicted partial ORFs and conserved motif. **A**) N-terminus ORF (derived from Unigene56418_All) with signal peptide (highlighted in red),10 predicted allatostatin peptides (highlighted in green), and amidated glycine (highlighted in light blue), separated by carboxyl-peptidase cleavage sites (underlined). **B**) Middle part ORF (derived from Unigene36127_All) with 8 predicted allatostatin peptides (highlighted in green), separated by carboxyl-peptidase cleavage sites (underlined). **C**) C- terminus ORF (derived from Unigene45628_All) with 4 predicted carcinustatin peptides (highlighted in green) and amidated glycine (highlighted in light blue), separated by carboxyl-peptidase cleavage sites (underlined). Asterisk indicates the stop codon. **D**) Type A allatostatin peptides conservation: 22 predicted neuropeptides of 8-10 aa in length derived from 3 putative partial transcripts with XXXX**Y**X**FGLamide** conserved.

Three transcripts were identified to putatively encode partial **type A allatostatin** precursors representing the N-terminus, middle region and C-terminus, with 248, 154 and 93 amino acids (aa), respectively (Table 1 and Fig. 1). The precursor N-terminus has a predicted signal peptide of 27 aa, followed by 10 predicted neuropeptides, separated by dibasic proteinase cleavage sites (Fig. 1A), while the middle and C-terminus contain 8 and 4 predicted neuropeptides (respectively), also separated by dibasic proteinase cleavage sites (Fig. 1B, C). The 22 predicted neuropeptides are 8 residues in length with **Y**X**FGLamide** highly conserved motif at the C-terminus of each peptide (Fig. 1D). Using BLAST of the mature neuropeptides individually, they were shown to have either high similarity, or, for most, exact identity to other type A allatostatins, primarily from decapod crustacean species, apart from two who were most similar to insect species. Most of the Eastern rock lobster putative type A allatostatin neuropeptides (17/22) had highest homology to type A allatostatin of the spiny lobster *Panulirus interruptus* (Table 2). All three type A allatostatin-encoding transcripts were found to have comparable expression levels with significantly higher expression in the brain, compared to the eyestalk (Table 3).

**Table 1:** Alphabetical list of predicted peptide precursors with transcript and ORF size and best BLAST hit.

Hormone	Transcript	Transcript size	ORF size	Comments	Best BLASTP result (Protein name [species] accession number)	E-Value
Allatostatins	>Unigene56418_All	955	248	A-type prepro-allatostatin, partial (N terminus)	allatostatin precursor protein [Panulirus interruptus] BAF64528	1.00E-115
	>Unigene36127_All	462	154	A-type prepro-allatostatin, partial (middle)	allatostatin precursor protein [Panulirus interruptus] BAF64528	1.00E-64
	>Unigene45628_All	1797	93	A-type prepro-allatostatin, partial (C terminus)	allatostatin precursor protein [Panulirus interruptus] BAF64528	6.00E-45
	>Unigene40422_All	704	152	B-type prepro-allatostatin, partial (N terminus)	B-type preproallatostatin II [Pandalopsis japonica] AFV91539	4.00E-21
	>Unigene25318_All	1537	135	B-type prepro-allatostatin, partial (C terminus)	B-type preproallatostatin II [Pandalopsis japonica] AFV91539	6.00E-44
	>CL2090.Contig2_All	3784	141	C-type prepro-allatostatin	C-type preproallatostatin [Pandalopsis japonica] AFV91540	1.00E-33
	>Unigene59348_All	1490	105	Insects prohormone-1	prohormone-1 [Apis mellifera] XP_001121443	5.00E-26
Bursicon α subunit	>CL593.Contig3_All	1228	142	prepro-Bursicon α 2	bursicon [Procambarus clarkii] ADY80040	3.00E-79
Corazonin	>Unigene32841_All	210	49	prepro-corazonin, partial	corazonin preprohormone [Daphnia pulex] ACJ05606	4.00E-06
CCAP (crustacean cardioactive peptide)	>Unigene1674_All	1107	139	prepro-CCAP	crustacean cardioactive peptide [Homarus gammarus] ABB46292	4.00E-62
CHH (crustacean hyperglycemic hormone)	>CL7809.Contig1_All	1021	135	prepro-CHH isoform B 1	prepro-crustacean hyperglycemic hormone isoform B [Nephrops norvegicus] AAQ22392	1.00E-60
	>CL7809.Contig3_All	1045	133	prepro-CHH isoform B 2	hyperglycemic hormone B [Homarus gammarus] ABA42180	8.00E-57
	>CL7809.Contig4_All	1576	112	prepro-CHH isoform B 3, partial (C terminus)	crustacean hyperglycemic hormone-like peptide precursor [Procambarus clarkii] ADZ98836	1.00E-40
	>Unigene30324_All	1453	126	prepro-CHH isoform B 4, partial (C terminus)	prepro-crustacean hyperglycemic hormone isoform B [Nephrops norvegicus] AAQ22392	8.00E-60
	>Unigene34312_All	1611	139	prepro-CHH, unspecified	hyperglycemic hormone [Pandalopsis japonica] AFG16932	5.00E-25
MIH/GIH (molt/gonad-inhibiting hormone)	>Unigene47171_All	679	115	prepro-MIH/GIH isoform A 1	prepro-gonad-inhibiting hormone isoform A [Macrobrachium nipponense] AEJ54622	4.00E-27
	>Unigene60521_All	1232	114	prepro-MIH/GIH isoform A 2	prepro-gonad-inhibiting hormone isoform A [Macrobrachium nipponense] AEJ54622	2.00E-26
	>Unigene58466_All	820	111	prepro-MIH/GIH isoform A 3	vitellogenesis inhibiting hormone [Homarus gammarus] ABA42181	3.00E-45
CFSH (crustacean female sex hormone)	>Unigene48118_All	1067	278	prepro-CFSH	crustacean female sex hormonoe, partial [Carcinus maenas] AEI72264	2.00E-08
DH (calcitonin-like diuretic hormone)	>CL8244.Contig2_All	1918	135	prepro-DH class 2	prepro-calcitonin-like diuretic hormone [Homarus americanus] ACX46386	2.00E-69
Eclosion hormone	>CL2590.Contig2_All	1584	82	prepro-Eclosion hormone 1	eclosion hormone [Amphibalanus amphitrite] AFK81936	2.00E-14
	>Unigene55076_All	757	86	prepro-Eclosion hormone 2	Eclosion hormone [Acromyrmex echinatior] EGI68318	4.00E-13
FLP (Myosuppressin)	>Unigene55051_All	819	100	prepro-FLP	prepro-myosuppressin [Homarus americanus] ACX46385	2.00E-40
Follistatin	>CL3958.Contig2_All	686	133	Follistatin-like	follistatin-like, partial [Nematostella vectensis] ABF61774	2.00E-15
	>Unigene49446	708	204	Follistatin-like, partial (N terminus)	hypothetical protein DAPPUDRAFT_303124 [Daphnia pulex] EFX89772	3.00E-41
Myostatin	>CL113.Contig2_All	1831	419	Myostatin	MSTN [Penaeus monodon] ADO34177	0
NPY (neuropeptide Y)	>Unigene30121_All	1287	104	prepro-NPF	neuropeptide Y [Lymnaea stagnalis] CAB63265	3.00E-09
Neuroparsin	>Unigene5705_All	1217	103	prepro-Neuroparsin	neuroparsin 1 precursor [Schistocerca gregaria] CAC38869	3.00E-12
	>CL2744.Contig6_All	1176	102	prepro-Neuroparsin 2	neuroparsin 1 precursor [Rhodnius prolixus] ACZ96369	7.00E-11
Orcokinin	>Unigene692_All	1343	205	prepro-Orcokinin	prepro-orcokinin II [Homarus americanus] ACD13197	2.00E-104
PDH (pigment dispersing hormone)	>CL7594.Contig2_All	430	79	prepro-PDH	pigment dispersing hormone related peptide precursor 79 - penaeid shrimp [Penaeus sp.] JC4756	2.00E-29
	>CL7594.Contig3_All	603	79	prepro-PDH	pigment dispersing hormone related peptide precursor 79 - penaeid shrimp [Penaeus sp.] JC4756	1.00E-23
Prohormone-3	>CL1958.Contig1_All	2238	196	prohormone-3	prohormone-3 [Apis mellifera] XP_001122204	1.00E-44
Prohormone-4	>Unigene19311_All	807	143	prohormone-4	prohormone-4-like [Acyrthosiphon pisum] XP_001951503	3E-86
RPCH (red pigment concentrating hormone)	>Unigene2547_All	1158	99	prepro-RPCH	red pigment concentrating hormone [Macrobrachium rosenbergii] ABV46765	2.00E-26
Sulfakinin	>Unigene25008_All	902	115	prepro-Sulfakinin	preprosulfakinin [Homarus americanus] ABQ95346	7E-53
Tachykinin	>CL7656.Contig2_All	2181	226	prepro-Tachykinin	preprotachykinin B [Panulirus interruptus] BAD06363	2.00E-143

**Table 2:** Alphabetical list of peptides and their best BLAST hit.

Hormone	Best BLAST hit	Accession number	Identity
Allatostatin A			
HNNYAFGLa	allatostatin precursor protein [Panulirus interruptus]	BAF64528	100% identity
TPDYAFGLa	allatostatin precursor protein [Panulirus interruptus]	BAF64528	100% identity
EGMYSFGLa	allatostatin precursor protein [Panulirus interruptus]	BAF64528	DGMYSFGLa
ADLFSFGLa	allatostatin precursor protein [Panulirus interruptus]	BAF64528	100% identity
SGNYNFGLa	allatostatin precursor protein [Panulirus interruptus]	BAF64528	100% identity
SQYAFGLa	A-type allatostatin [Amphibalanus amphitrite]	AFK81929	100% identity
SKLYSFGLa	FGLa-related allatostatin [Nilaparvata lugens]	BAO00953	QKLYSFGLa
NRQYSFGLa	allatostatin precursor protein [Panulirus interruptus]	BAF64528	100% identity
SQQYAFGLa	type-a prepro-allatostatin [Macrobrachium nipponense]	AEX86939	100% identity
PRNYAFGLa	allatostatin precursor protein [Panulirus interruptus]	BAF64528	100% identity
PTAYSFGLa	allatostatin precursor protein [Panulirus interruptus]	BAF64528	PTTYSFGLa
TASYGFGLa	allatostatin precursor protein [Panulirus interruptus]	BAF64528	100% identity
SDLYDNDLGRSYDFGL	allatostatin precursor protein [Panulirus interruptus]	BAF64528	SDSYDNGLGRRSYDFGL
SGPYAFGLa	allatostatin precursor protein [Panulirus interruptus]	BAF64528	100% identity
GGPYAFGLa	type-a pre-proallatostatin [Macrobrachium rosenbergii]	AAY82901	100% identity
ADLYSFGLa	allatostatin precursor protein [Panulirus interruptus]	BAF64528	100% identity
ADPYSFGLa	allatostatin precursor protein [Panulirus interruptus]	BAF64528	100% identity
AGQYSFGLa	allatostatin precursor protein [Panulirus interruptus]	BAF64528	100% identity
AGPYSFGLa	allatostatin precursor protein [Panulirus interruptus]	BAF64528	100% identity
EDSPASDAYTL	allatostatin precursor protein [Panulirus interruptus]	BAF64528	EDSSASDPYIL
SGSYSFGLa	type-a prepro-allatostatin [Macrobrachium nipponense]	AEX86939	100% identity
AGPYSFGLa	allatostatin precursor protein [Panulirus interruptus]	BAF64528	100% identity
Allatostatin B			
TDWSSMHGTWa	B-type preproallatostatin II [Pandalopsis japonica]	AFV91539	ADWSSMRGTWa
PDLLQAPLQAVGD	Na		
GNWDKFHGSWa	B-type preproallatostatin II [Pandalopsis japonica]	AFV91539	ANWNKFQGSWa
AEEIQAAED	Na		
ADWNKFHGSWa	Na		
GDEFASPELETTED	Na		
ANWNKFHGSWa	B-type preproallatostatin II [Pandalopsis japonica]	AFV91539	ANWNKFQGSWa
GDDLVDAEL	Na		
DWSSLQGTWa	B-type preproallatostatin I, partial [Pandalopsis japonica]	AFV91539	GWSSLQGSWa
DWNNLHGAWa	B-type preproallatostatin I, partial [Pandalopsis japonica]	AFV91539	AWKNLHGAWa
SPDWNSLRGAWa	B-type preproallatostatin I, partial [Pandalopsis japonica]	AFV91539	SGDWNSLRGAWa
APDWAQFRGSWa	B-type preproallatostatin I, partial [Pandalopsis japonica]	AFV91539	DGDWSQFRGSWa
VPDEVNETAAHQA	Na		
Allatostatin C			
ALGEEQLQEEAAKS	Na		
MFAPLSGLPGELPTI	C-type preproallatostatin [Pandalopsis japonica]	AFV91540	LFAPLSGLPGEIPTM
QIRYHQCYFNPISCF	C-type preproallatostatin [Pandalopsis japonica]	AFV91540	QIRYRQCYFNPISCF
Hormone-1			
SYWKQCAFNAVSCFa	prohormone-1 isoform X2 [Apis mellifera]	XP_006570429	100% identity
Bursicon alpha subunit	bursicon [Procambarus clarkii]	ADY80040	90% identity
Corazonin			
TFQYSRGWTNa	Pro-corazonin [Harpegnathos saltator]	EFN88292	100% identity
Crustacean cardioactive peptide	crustacean cardioactive peptide [Homarus gammarus]	ABB46292	81% identity in 75% cover
Crustacean female sex hormone	crustacean female sex hormonoe, partial [Carcinus maenas]	AEI72264	26% identity
Crustacean hyperglycemic hormone (CHH) isoform B1	prepro-crustacean hyperglycemic hormone isoform B [Nephrops norvegicus]	AAQ22392	82% identity
CHH isoform B2	crustacean hyperglycemic hormone isoform 2 [Rimicaris kairei]	ACS35347	81% identity
CHH isoform B3	CHH-like protein precursor [Procambarus clarkii]	AF474408	64% identity
CHH isoform B4	prepro-crustacean hyperglycemic hormone isoform B [Nephrops norvegicus]	AAQ22392	85% identity
CHH unspecified	hyperglycemic hormone [Pandalopsis japonica]	AFG16932	59% identity
Molt inhibiting hormone (MIH) isoform 1	Molt-inhibiting hormone [Orconectes limosus]	P83636	55% identity
MIH isoform 2	Probable molt-inhibiting hormone [Jasus lalandi]	P83220	70% identity
MIH isoform 3	Vitellogenesis inhibiting hormone [Homarus gammarus]	ABA42181	72% identity
Diuretic hormone	prepro-calcitonin-like diuretic hormone [Homarus americanus]	ACX46386	90% identity
Eclosion hormone isoform 1	eclosion hormone 2 [Nilaparvata lugens]	BAO00951	62% identity
Eclosion hormone isoform 2	eclosion hormone 1 [Nilaparvata lugens]	BAO00950	49% identity
FLP (myosupressin)	myosuppressin-like neuropeptide precursor [Procambarus clarkii]	BAG68789	86% identity
Follystatin isoform 1	*follistatin-like, partial [Nematostella vectensis]*	ABF61774	54% identity
Follystatin isoform 2	*follistatin-related protein 1 isoform 1 [Odobenus rosmarus divergens]*	XP_004403583	38% identity
Myostatin	MSTN [Penaeus monodon]	ADO34177	65% identity
Neuropeptide Y	*neuropeptide Y [Lymnaea stagnalis]*	CAB63265	57% identity
Neuroparsin isoform 1	neuroparsin [Jasus lalandii]	AHG98659	97% identity
Neuroparsin isoform 2	neuroparsin [Jasus lalandii]	AHG98659	48% identity
Orcokinin			
FDAFTTGFGHSKR	Orcokinin [Procambarus clarkii]	Q9NL83	100% identity
NFDEIDRSGFAFAKK	Orcokinin [Procambarus clarkii]	Q9NL83	NFDEIDRSGFGFAKK
NFDEIDRAGLGFAKR	prepro-orcokinin II [Homarus americanus]	ACD13197	NFDEIDRSGFGFNKR
NFDEIDRSGFGFNKR	prepro-orcokinin II [Homarus americanus]	ACD13197	100% identity
NFDEIDRAGLGFHKR	prepro-orcokinin II [Homarus americanus]	ACD13197	NFDEIDRSGFGFHKR
NFDEIDRSGFGFNKR	prepro-orcokinin II [Homarus americanus]	ACD13197	100% identity
NFDEIDRTGFGFHKR	Orcokinin [Procambarus clarkii]	Q9NL83	100% identity
DYDGVYPDKR	prepro-orcokinin II [Homarus americanus]	ACD13197	DYD-VYPEKR
NFDEIDRAGFGFVKR	prepro-orcokinin II [Homarus americanus]	ACD13197	NFDEIDRSGFGFVKR
AFGPRDISNLYKR	prepro-orcokinin II [Homarus americanus]	ACD13197	VYGPRDIANLYKR
NFDEIDRSGFGFVRR	prepro-orcokinin II [Homarus americanus]	ACD13197	100% identity
Pigment dispersing hormone			
NAELINSILGLPKVMNDAa	Pigment-dispersing hormone [Uca pugilator]	P08871	NSELINSILGLPKVMNDAa
NAELINSLLGIPKVMSDAa	Pigment-dispersing hormone [Litopenaeus vannamei]	P91963	NSELINSLLGIPKVMNDAa
Hormone-3	prohormone-3 [Apis mellifera]	XP_001122204	43% identity
Hormone-4	prohormone-4-like [Acyrthosiphon pisum]	XP_001951503	89% identity
Red pigment concentrating hormone	Red pigment-concentrating prohormone [Callinectes sapidus]	Q23757	63% identity
Sulfakinin			
EFDEYGHMRFa	preprosulfakinin [Homarus americanus]	ABQ95346	100% identity
SGGEYDDYGHLRFa	preprosulfakinin [Homarus americanus]	ABQ95346	GGGEYDDYGHLRFa
Tachykinin			
APSGFLGMRa	preprotachykinin [Procambarus clarkii]	BAC82426	100% identity

Best BLAST hit shows arthropods that are not decapod crustaceans (underlined) and non-arthropods (*italicized and underlined*). Identity of proteins is given as percentage and peptides as sequence with non-identical aa underlined (amidation is noted by 'a').

**Table 3:** Alphabetical list of peptide precursors with RPKM quantity in male and female brain and eyestalk.

Hormone	Transcript	Comments	M BR	F BR	M ES	F ES	BR	ES
Allatostatins	>Unigene56418_All	A-type prepro-allatostatin, partial (N terminus)	34.32	35.6	16.05	13.42	**34.96**	14.74
	>Unigene36127_All	A-type prepro-allatostatin, partial (middle)	34.14	35.77	11.78	13.75	**34.96**	12.77
	>Unigene45628_All	A-type prepro-allatostatin, partial (C terminus)	32.56	38.8	13.85	13.49	**35.68**	13.67
	>Unigene40422_All	B-type prepro-allatostatin, partial (N terminus)	39.12	40.22	52.4	60.14	39.67	**56.27**
	>Unigene25318_All	B-type prepro-allatostatin, partial (C terminus)	46.66	54.5	57.75	64.03	50.58	*60.89*
	>CL2090.Contig2_All	C-type prepro-allatostatin	11.08	11.04	3.22	3.42	**11.06**	3.32
	>Unigene59348_All	Insects prohormone-1	527.56	565.95	379.48	353.88	**546.76**	366.68
Bursicon α subunit	>CL593.Contig3_All	prepro-Bursicon α 2	2.07	2.06	0	0	**2.07**	0.00
Corazonin	>Unigene32841_All	prepro-corazonin, partial	*0.54*	0	14.64	*20.12*	0.27	**17.38**
CCAP (crustacean cardioactive peptide)	>Unigene1674_All	prepro-CCAP	30.9	28.86	*81.84*	54.44	29.88	**68.14**
CHH (crustacean hyperglycemic hormone)	>CL7809.Contig1_All	prepro-CHH isoform B 1	0.12	*0.46*	309.12	*450.84*	0.29	**379.98**
	>CL7809.Contig3_All	prepro-CHH isoform B 2	0.04	*0.19*	127.88	*238.64*	0.12	**183.26**
	>CL7809.Contig4_All	prepro-CHH isoform B 3, partial (C terminus)	0.57	*0.7*	435.45	*652.06*	0.64	**543.76**
	>Unigene30324_All	prepro-CHH isoform B 4, partial (C terminus)	0	0	2.2	*3.41*	0.00	**2.81**
	>Unigene34312_All	prepro-CHH, unspecified	3.47	*5.92*	4.74	*5.36*	4.70	5.05
MIH/GIH (molt/gonad inhibiting hormone)	>Unigene47171_All	prepro-MIH/GIH isoform A 1	0	0	2.51	*4.65*	0.00	**3.58**
	>Unigene60521_All	prepro-MIH/GIH isoform A 2	0	0.13	297.72	*408.18*	0.07	**352.95**
	>Unigene58466_All	prepro-MIH/GIH isoform A 3	0.05	0	4.41	*8.33*	0.03	**6.37**
CFSH (crustacean female sex hormone)	>Unigene48118_All	prepro-CFSH	0	0	*6.86*	5.32	0.00	**6.09**
DH (calcitonin-like diuretic hormone)	>CL8244.Contig2_All	prepro-DH class 2	*78.61*	70.11	*66.36*	61.51	*74.36*	63.94
Eclosion hormone	>CL2590.Contig2_All	prepro-Eclosion hormone 1	*3.9*	3.01	*49.2*	29.2	3.46	**39.20**
	>Unigene55076_All	prepro-Eclosion hormone 2	0	0	0	*0.11*	0.00	*0.06*
FLP (Myosuppressin)	>Unigene55051_All	prepro-FLP	56.18	*65*	*58.99*	48.62	*60.59*	53.81
Follistatin	>CL3958.Contig2_All	Follistatin-like	0.18	0.29	0.06	0.06	0.24	0.06
	>Unigene49446	Follistatin-like, partial (N terminus)	0	0.06	0	0	0.03	0.00
Myostatin	>CL113.Contig2_All	Myostatin	4.24	5.19	13.07	13.52	4.72	**13.30**
NPY (neuropeptide Y)	>Unigene30121_All	prepro-NPF	3.08	2.91	47.64	46.86	3.00	**47.25**
Neuroparsin	>Unigene5705_All	prepro-Neuroparsin	428.86	*665.33*	*462.26*	370.29	*547.10*	416.28
	>CL2744.Contig6_All	prepro-Neuroparsin 2	*12.06*	6.44	14.2	*17.83*	9.25	*16.02*
Orcokinin	>Unigene692_All	prepro-Orcokinin	*72.2*	54.85	52.4	48.95	*63.53*	50.68
PDH (pigment dispersing hormone)	>CL7594.Contig2_All	prepro-PDH	*8.55*	0.18	150.22	144.9	4.37	**147.56**
	>CL7594.Contig3_All	prepro-PDH	*2.98*	0.33	70.95	62.33	1.66	**66.64**
Prohormone-3	>CL1958.Contig1_All	prohormone-3	*44.39*	23.14	60.68	60.61	33.77	**60.65**
Prohormone-4	>Unigene19311_All	prohormone-4	*51.09*	34.79	*28.36*	19.2	**42.94**	23.78
RPCH (red pigment concentrating hormone)	>Unigene2547_All	prepro-RPCH	12.84	15.22	48.32	52.63	14.03	**50.48**
Sulfakinin	>Unigene25008_All	prepro-Sulfakinin	*9.88*	0.66	*4.7*	3.01	*5.27*	3.86
Tachykinin	>CL7656.Contig2_All	prepro-Tachykinin	365.98	*457.61*	*100.12*	88.78	**411.80**	94.45

RPKM: number of reads mapped to the transcript per kilobase per million reads in the total library; M: male; F: female; BR: brain; ES: eyestalk. *Italicized and underlined* are values with a non statistically significant difference, **bold and underlined** are values with statistically significant difference, as calculated by ANOVA in Partek GS (p<0.05).

**Figure 2:**
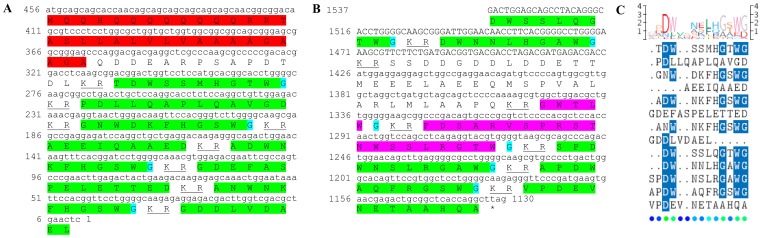
Type B allatostatin precursors predicted partial ORFs and conserved motif. **A**) N- terminus ORF (derived from Unigene40422_All) with signal peptide (red) and 8 predicted allatostatin peptides (green) with amidated glycine (light blue), separated by carboxyl-peptidase cleavage sites (underlined). **B**) C- terminus ORF (derived from Unigene25318_All) with 5 predicted allatostatin peptides (green), separated by carboxyl-peptidase cleavage sites (underlined). Asterisk indicates the stop codon. **C**) Type B allatostatin peptides conservation: 13 predicted neuropeptides of 9-14 aa in length derived from 2 putative partial transcripts with XX**DW**XXXXXX**G**X**Wamide** conserved.

Two transcripts were identified to putatively encode partial **type B allatostatin** precursors representing the N-terminus and C-terminus, with 152 and 135 aa, respectively (Table 1 and Fig. 2). The N-terminus has a predicted signal peptide of 33 aa, followed by 8 predicted neuropeptides, separated by dibasic proteinase cleavage sites (Fig. 2A), while in the C-terminus there are 5 predicted neuropeptides, separated by dibasic proteinase cleavage sites (Fig. 2B). The 13 predicted neuropeptides are 9–14 aa in length with XX**DW**XXXXXX**G**X**Wamide** conserved motif (Fig. 2C). BLAST identified 7 of the above 13 neuropeptides in type B allatostatin of the caridean shrimp *Pandalus japonica*, while the other 6 appear to be novel (Table 2). Both transcripts were found to have comparable expression levels with significantly higher expression of the N-terminus in the eyestalk, compared to the brain (Table 3).

**Figure 3:**
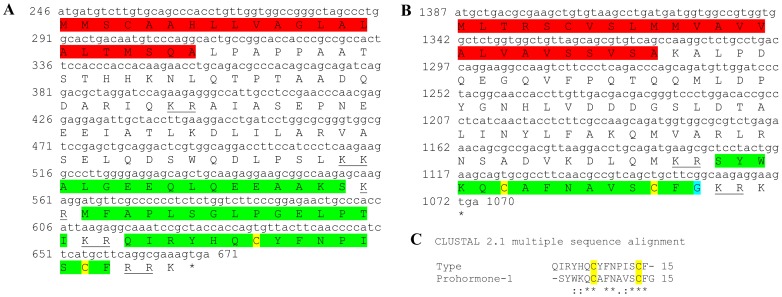
Type C allatostatin and prohormone-1 precursor predicted ORFs and conserved peptide. **A**) A complete ORF (derived from CL2090.Contig2_All) of type C allatostatin precursor with a signal peptide (red) and 3 predicted allatostatin peptides (green) with an amidated glycine (light blue), separated by carboxyl-peptidase cleavage sites (underlined). **B**) A complete ORF (derived from Unigene59348_All) of prohormone-1 with a signal peptide (red) and a predicted allatostatin peptide (green), separated by carboxyl-peptidase cleavage sites (underlined). Two conserved cysteine residues in the last allatostatin peptide of each sequence are highlighted in yellow. Asterisk indicates the stop codon. **C**) Amino acid alignment between the conserved peptides of C type allatostatin and prohormone-1.

One transcript was identified to putatively encode a complete **type C allatostatin** precursor with 141 aa, starting with a signal peptide of 22 aa, followed by 3 putative neuropeptides, separated by dibasic proteinase cleavage sites (Fig. 3A). The predicted neuropeptides are 14–15 aa in length with no homology between them. The peptide at the precursor C-terminus has two cysteine residues characteristic of other allatostatins (Fig. 3A). Two of the three neuropeptides shared high identity with type C allatostatin identified in *P. japonica* (Table 2). The transcript level was found to be significantly higher in the brain compared to the eyestalk (Table 3). Another transcript was identified to putatively encode a complete prohormone-1 with 105 aa, starting with a signal peptide of 25 aa, followed by 1 putative neuropeptide, separated by dibasic proteinase cleavage sites (Fig. 3B). The putative neuropeptide in prohormone-1 shares a conserved motif (**QC**X**FN**XX**SCF**) with the last putative peptide in the type C allatostatin (Fig. 3C), and is identical to the neuropeptide encoded by prohormone-1 of insects (Table 2). While like allatostatin type C, prohormone-1 has a significantly higher expression in the brain compared with eyestalk, the overall expression of prohormone-1 is one order of magnitude higher compared to all other allatostatins (Table 3).

### Bursicon alpha subunit

**Figure 4:**
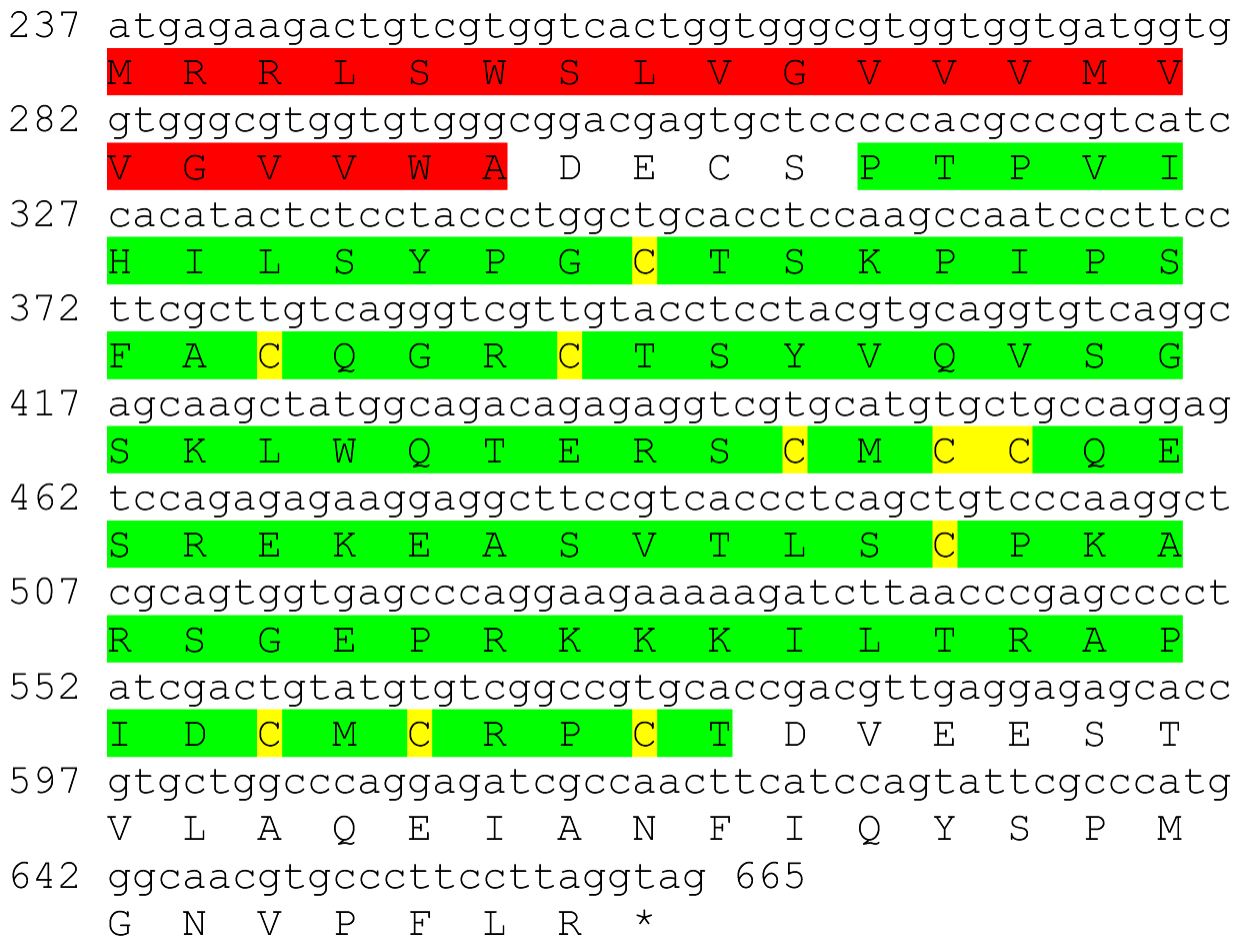
Bursicon alpha subunit precursor predicted ORF. A complete ORF (derived from CL593.Contig3_All) of bursicon alpha subunit precursor with a signal peptide (red) and a predicted C-terminal cysteine knot-like domain (green). Ten conserved cysteine residues are highlighted in yellow. Asterisk indicates the stop codon.

One transcript was identified to putatively encode a complete bursicon alpha subunit precursor with 142 aa, starting with a 25 aa signal peptide, followed by a predicted C-terminal cysteine knot-like domain of 89 aa which contains ten conserved cysteine residues (Table 1 and Fig. 4). The mature hormone share up to 90% identity with bursicon alpha subunit identified in other decapod crustacean species (Table 2). The level of expression is very low in the brain and not evident in the eyestalk (Table 3).

### Corazonin

**Figure 5:**
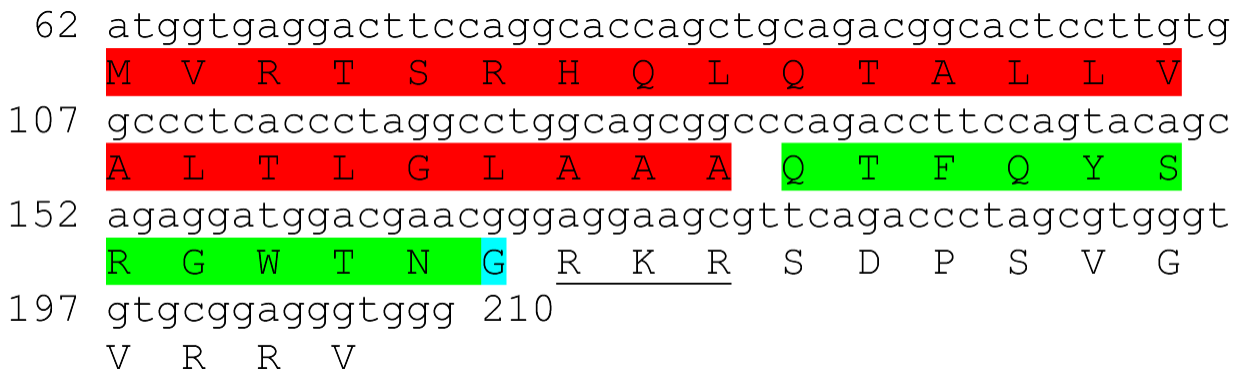
Corazonin predicted precursor ORF. A partial ORF (derived from Unigene32841_All) of the N- terminus of corazonin precursor with a signal peptide (red) and a conserved peptide (green) with an amidated glycine (light blue), followed by a carboxyl-peptidase cleavage site.

One transcript was identified to putatively encode 49 aa of the N-terminus of the corazonin precursor, starting with a 24 aa long signal peptide followed by a 11 aa conserved peptide (identical to corazonin peptides of insects; Table 2) followed by a carboxyl-peptidase cleavage site (Table 1 and Fig. 5). Corazonin expression was found to be almost exclusive to the eyestalk with slight higher levels in females (Table 3).

### Crustacean cardioactive peptide (CCAP)

**Figure 6:**
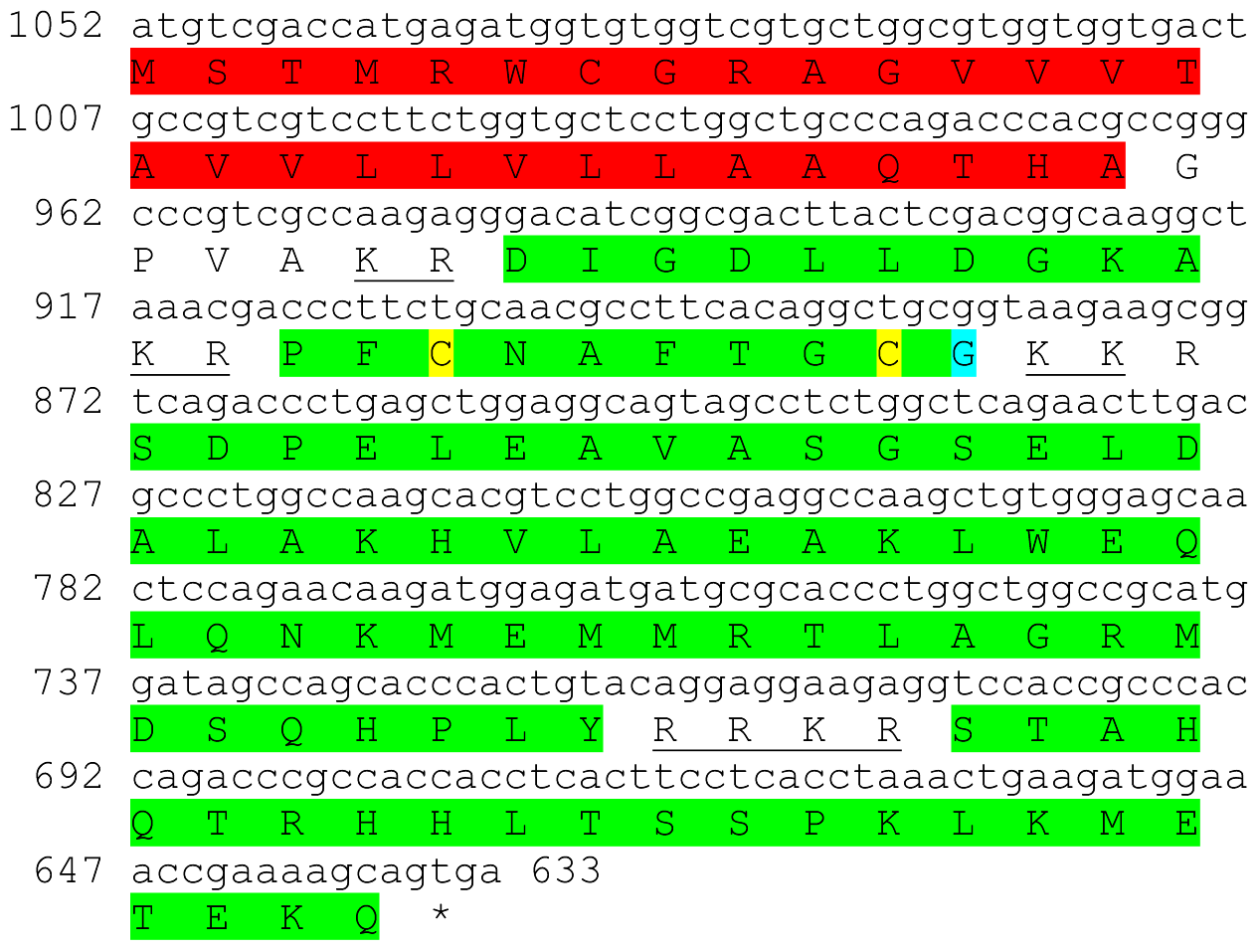
Crustacean cardioactive peptide (CCAP) predicted ORF. A complete ORF (derived from Unigene1674_All) of CCAP with a signal peptide (red) and four predicted peptides (green) with an amidated glycine (light blue), separated by carboxyl-peptidase cleavage sites. Two conserved cysteine residues are highlighted in yellow. Asterisk indicates the stop codon.

One transcript was identified to putatively encode a complete 139 aa open reading frame (ORF) of CCAP precursor starting with a 29 aa signal peptide followed by four predicted peptides (10, 9, 52 and 23 aa in length), separated by carboxyl-peptidase cleavage sites. One of those peptides is highly conserved and contains two cysteine residues predicted to form a disulfide bridge and is amidated (Table 1 and Fig. 6). The highest identity level of the entire ORF, excluding the signal peptide was 81%, with another decapod crustacean CCAP, covering 75% of the ORF (Table 2). The transcript encoding CCAP had significantly higher expression in the eyestalk compared with the brain, with a higher expression in male eyestalk (Table 3).

### Crustacean hyperglycemic hormone (CHH)

**Figure 7:**
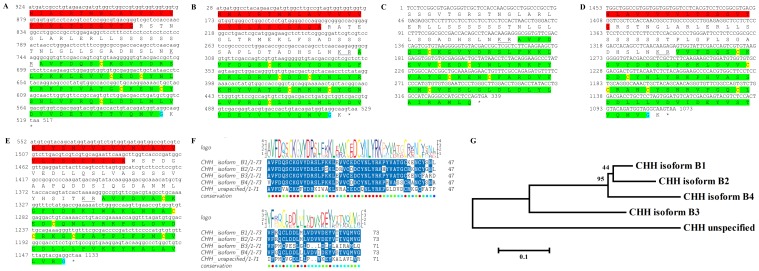
Crustacean hyperglycemic hormone (CHH) precursors predicted complete and partial ORFs with high similarity levels. A–E) Complete and partial CHH isoforms 1–5 (derived from CL7809.Contig1_All, CL7809.Contig3_All, CL7809.Contig4_All, Unigene30324_All and Unigene34312_All) with signal peptides (red) and predicted CHH domains (green) with an amidated glycine (light blue), preceded by carboxyl-peptidase cleavage sites (underlined). Six conserved cysteine residues predicted to form 3 disulfide bridges are highlighted in yellow. Asterisk indicates the stop codon. F) CHH domains conservation: the 71–73 aa domains show high level of similarity with each other. G) CHH domains phylogenetic tree showing similarity levels are highest between isoforms B1 and B2, followed by isoform B4, then isoform B3 and furthest is the unspecified CHH isoform. Scale bar represents number of substitutions per site.

Five transcripts were identified to putatively encode three complete and two partial CHH peptide precursors with 112–139 aa (Table 1 and Fig. 7). All three complete sequences start with a predicted signal peptide of 25–26 aa. One partial sequence has part of the signal peptide (16 aa). All 5 sequences have a CHH-conserved domain of 71–73 aa, preceded by a carboxyl-peptidase cleavage site. The 6 cysteine residues predicted to give rise to 3 disulfide bridges are all aligned between the 5 sequences (Fig. 7A–E). Overall the sequence similarity between the CHH domains is high with up to 89% identity between isoforms B1 and B2 (Fig. 7F, G). Compared with previously described CHHs, identity of the mature hormone was between 59%–85% (Table 2). Isoforms B1-3 had the highest expression of all five transcripts, and found almost exclusively in the eyestalk, while isoform B4 had much lower expression (two orders of magnitude) only in the eyestalk. The unspecified isoform had equivalent expression to that of isoform B4 in both the eyestalk and the brain. Interestingly, all five isoforms had higher levels in females compared with males (Table 3).

### Molt/Gonad-inhibiting hormone (MIH/GIH)

**Figure 8:**
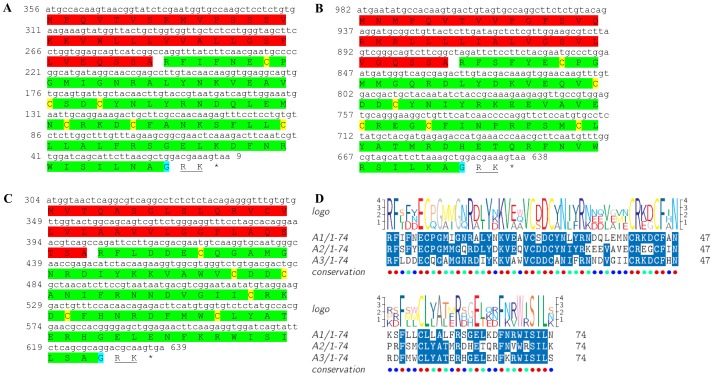
MIH predicted complete and partial ORFs with intermediate similarity. **A–C**) Complete MIH isoforms 1–3 (derived from Unigene47171_All, Unigene60521_All and Unigene58466_All) with signal peptides (red) and predicted MIH domains (green) with an amidated glycine (light blue). Six conserved cysteine residues predicted to form 3 disulfide bridges are highlighted in yellow. Asterisk indicates the stop codon. **D**) MIH domains conservation: the 74 aa domains show intermediate level of similarity with each other.

Three transcripts were identified to putatively encode three complete MIH/GIH peptide precursors with 111–115 aa (Table 1 and Fig. 8). All three sequences start with a predicted signal peptide of 33–37 aa followed by an MIH-conserved domain of 74 aa. The 6 cysteine residues predicted to give rise to 3 disulfide bridges are all aligned between all 3 sequences (Fig. 8A–C). Overall, the sequence similarity between the MIH domains is lower than the CHH isoforms with 53%–54% identity (Fig. 8D). Compared with previously described MIHs/GIHs, identity of the mature hormone was between 55%–72% (Table 2). All 3 putative MIH transcripts were found to be specifically expressed in the eyestalk with isoform A2 showing highest expression. Similar to CHH, all three MIH isoforms showed higher expression levels in females compared with males (Table 3).

### Crustacean female Sex hormone (CFSH)

**Figure 9:**
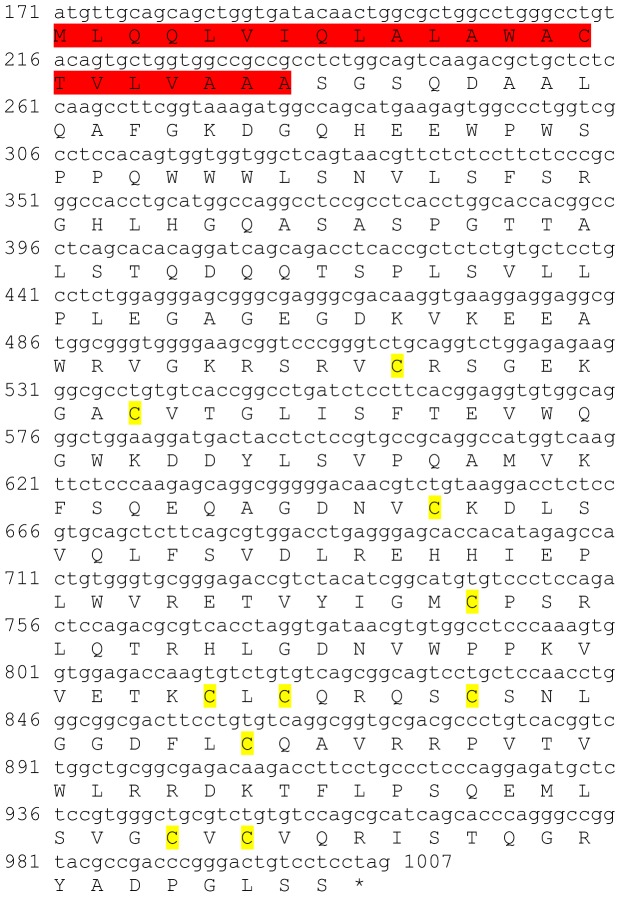
CFSH precursor predicted complete ORF. A complete CFSH like peptide (derived from Unigene48118_All) with a signal peptide (red) and 10 conserved cysteine residues predicted to form 5 disulfide bridges (yellow). Asterisk indicates the stop codon.

One transcript was identified to putatively encode a complete CFSH peptide precursor with 278 aa (Table 1 and Fig. 9). The sequence starts with a 22 aa signal peptide and contains 10 conserved cysteine residues predicted to form 5 disulfide bridges (Fig. 9), although the overall identity of the mature hormone does not exceed 26% with other decapod crustaceans (Table 2). CFSH was found to be specifically expressed in the eyestalk, with equivalent expression in both males and females (Table 3).

### Diuretic hormone (DH)

**Figure 10:**
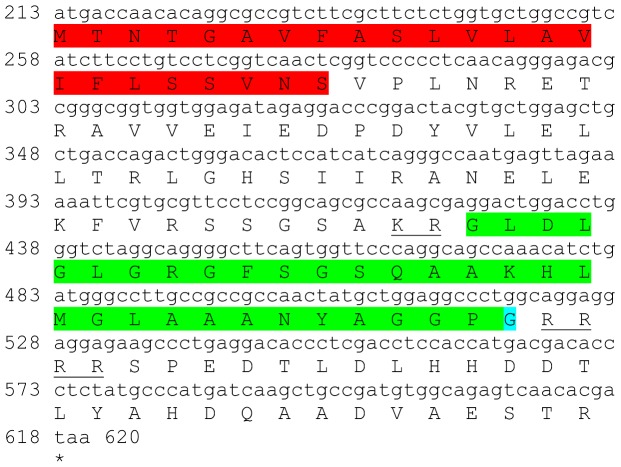
DH precursor predicted complete ORF. A complete DH-like peptide precursor (derived from CL8244.Contig1_All) with a signal peptide (red) and aconserved peptide (green) with an amidated glycine (light blue), bordered by carboxyl-peptidase cleavage sites.Asterisk indicates the stop codon.

One transcript was identified to putatively encode a complete DH peptide precursor with 135 aa (Table 1 and Fig. 10). The sequence starts with a 23 aa signal peptide and the active 31-residue DH peptide is released using dibasic proteinase cleavage sites. This peptide shared 90% identity with a clawed lobster DH (Table 2). The transcript is expressed in both brain and eyestalk with a non significant higher level in brain and in males (Table 3).

### Eclosion hormone

**Figure 11:**
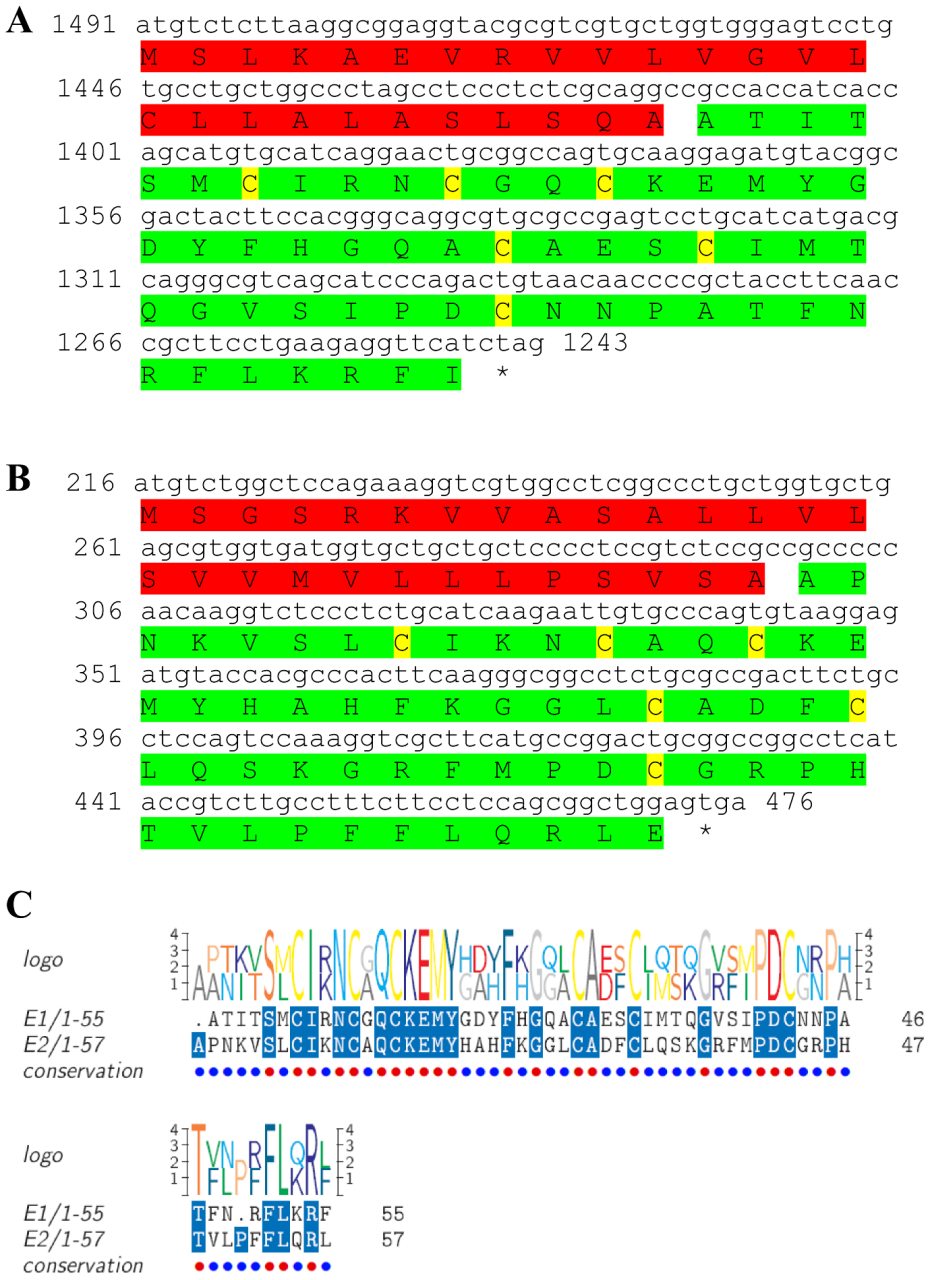
Eclosion hormone precursor predicted ORFs and conserved peptide. **A, B**) Complete ORFs (derived from CL2590.Contig2_All and Unigene55076_All) of eclosion hormone precursor each starting with a signal peptide (red) followed by an eclosion hormone domain (green) with 6 conserved cysteine residues (yellow). Asterisk indicates the stop codon. **C**) Amino acid alignment between the eclosion hormone domains.

Two transcripts were identified to putatively encode complete isoforms of the eclosion hormone precursor (Table 1 and Fig. 11) with 82 and 86 aa, each starting with a signal peptide of 26–28 aa, followed by 55–57 aa eclosion hormone domains each containing 6 conserved cysteine residues predicted to form 3 disulfide bridges (Fig. 11A, B). Other than the cysteine residues, the similarity level between the two eclosion hormone domains is intermediate, with 47% identity (Fig. 11C). Compared to other eclosion hormones, identity of *S. verreauxi* eclosion was 49%–62% with insect eclosion hormones (Table 2). The first isoform had a significantly higher expression in the eyestalk compared with the brain, and higher expression in males compared with females. The second isoform showed only a basal expression in the female eyestalk (Table 3).

### Follistatin

**Figure 12:**
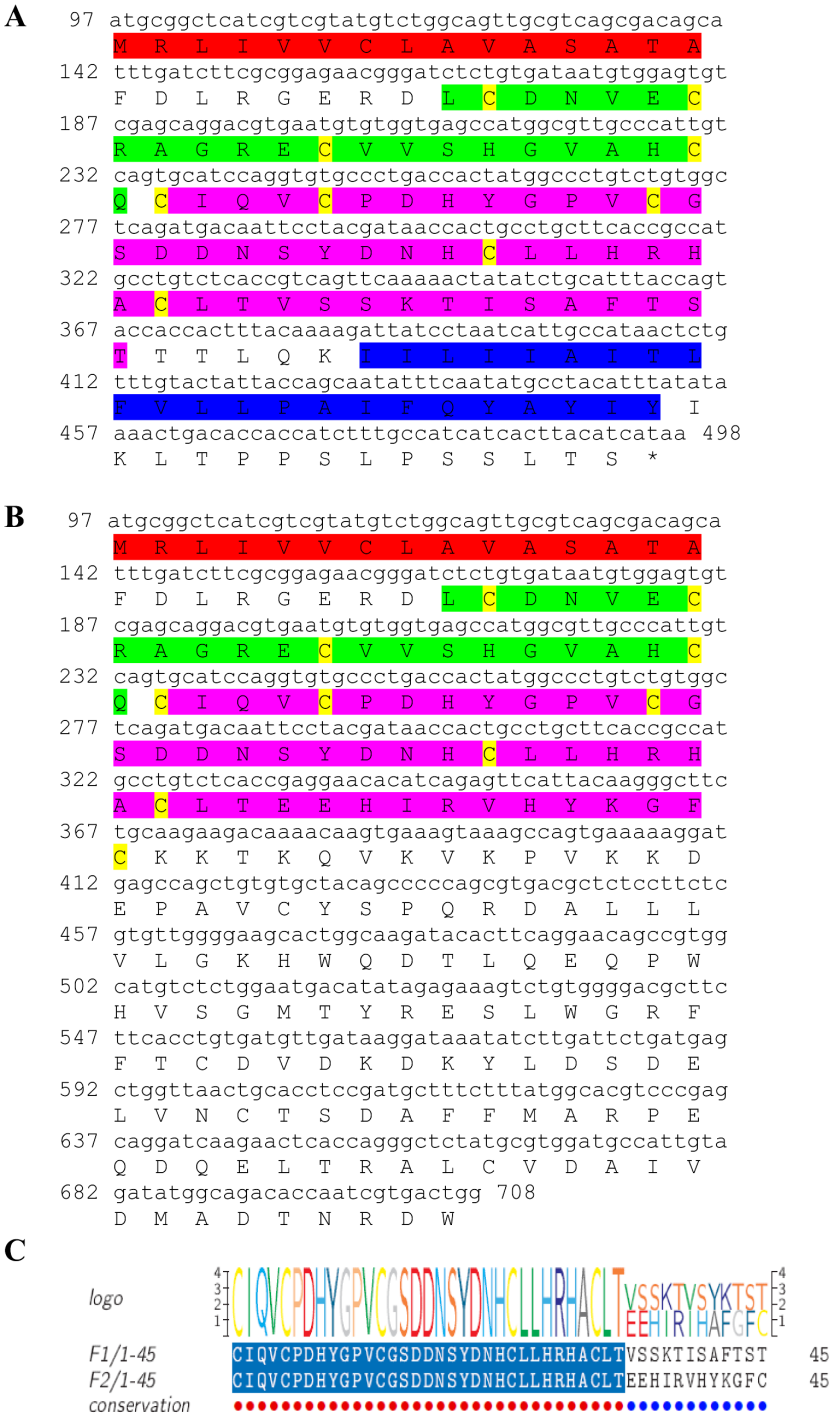
Follistatin precursors predicted ORFs and conserved peptide. **A, B**) Complete and a partial follistatin precursor predicted ORFs (derived from CL3958.Contig2_All and Unigene49446_All) each starting with a signal peptide (red) followed by an identical follistatin domain (green) with 4 conserved cysteine residues (yellow), followed by a kazal-type serine protease inhibitor domain (pink) with 5–6 cysteine residues (yellow). The complete, shorter isoform (A) ends with a predicted transmembrane domain (blue). Asterisk indicates the stop codon. **C**) Amino acid alignment between the kazal-type domains.

Two transcripts were identified to putatively encode a complete (133 aa) and a partial (204 aa) isoforms of the follistatin precursor (Table 1 and Fig. 12), each starting with a signal peptide of 15 aa, followed by identical 23 aa follistatin domains each containing 4 conserved cysteine residues predicted to form 2 disulfide bridges (Fig. 12A, B). In each predicted peptide, the follistatin domain is followed by a 45 aa kazal-type serine protease inhibitor domain whose N-terminus is identical between the isoforms with 5 cysteine residues and the C-terminus contains 2 additional cysteine residues in the partial isoform (Fig. 12C). The shorter, yet complete follistatin-like isoform ends with a 23 aa predicted transmembrane region. The mature hormones showed identity of 38%–54% to a cnidarians and a mammalian species' follistatins (Table 2). The first transcript had a very low expression in all tissues and the second transcript had very low expression and was exclusively found in the female brain (Table 3).

### Myostatin

**Figure 13:**
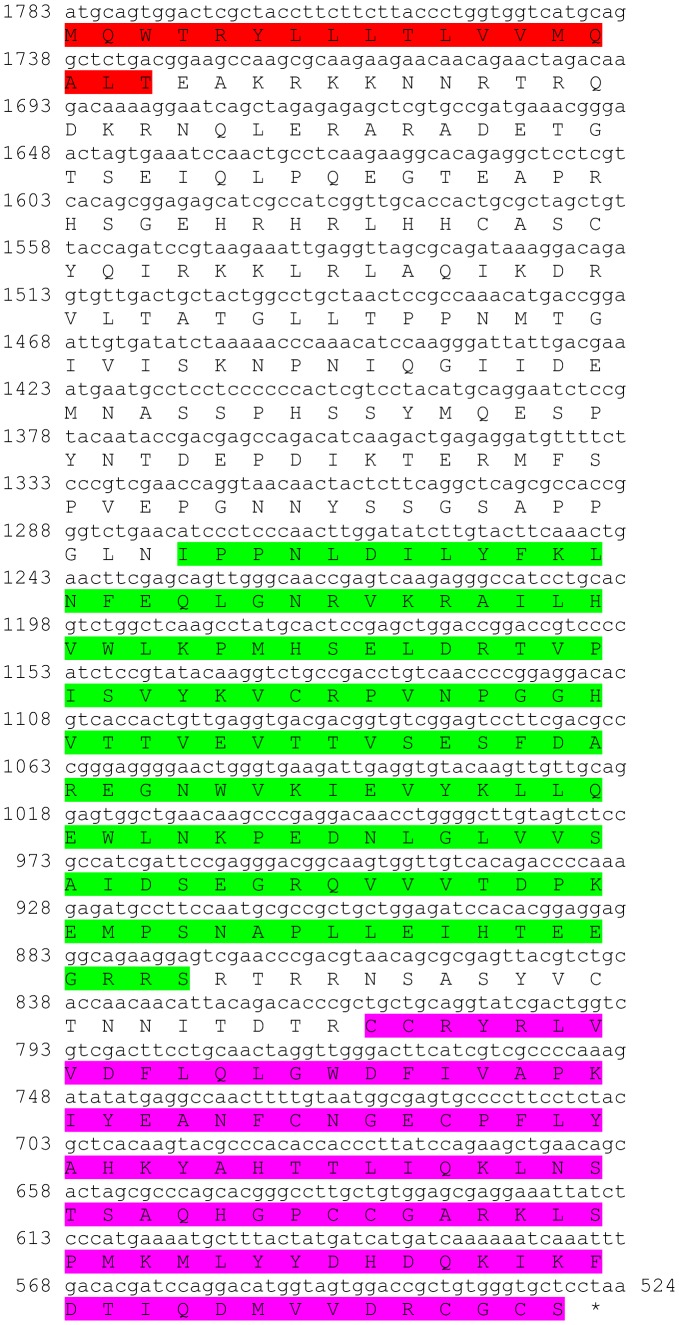
Myostatin precursor predicted ORF. A complete myostatin predicted ORF (derived from CL113.Contig2_All) starting with a signal peptide (red) followed by a TGF-beta propeptide domain (green), followed by another TGF-beta domain domain (pink). Asterisk indicates the stop codon.

One transcript was identified to putatively encode a complete 419 aa ORF of a myostatin precursor, starting with a 18 aa signal peptide, followed by a 136 aa TGF-beta propeptide domain, followed by another 96 aa TGF-beta domain (Table 1 and Fig. 13). The mature hormone showed 65% identity with another decapod crustacean myostatin (Table 2). Myostatin showed significantly higher expression in the eyestalk compared to the brain (Table 3).

### Myosupressin

**Figure 14:**
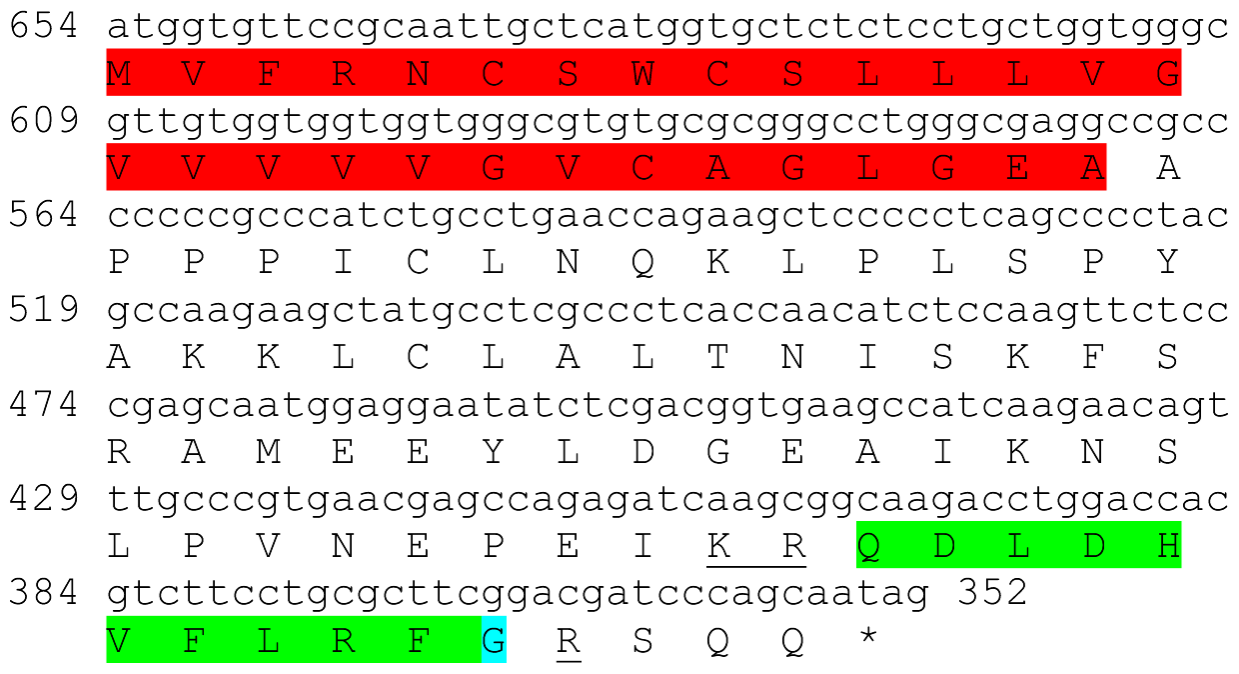
Myosupressin precursor predicted complete ORF. A complete Myosupressin peptide precursor (derived from Unigene55051_All) with a signal peptide (red) and a conserved peptide (green) with an amidated glycine (light blue), bordered by carboxyl-peptidase cleavage sites. Asterisk indicates the stop codon.

One transcript was identified to putatively encode a complete myosupressin peptide precursor with 100 aa (Table 1 and Fig. 14). The sequence starts with a 29 aa signal peptide and the active 10-residue myosupressin peptide is released using dibasic and arginine proteinase cleavage sites. Overall the prohormone showed 86% identity with myosupressin of the penaeid shrimp *Penaeus monodon* (Table 2). Myosupressin showed similar expression in the eyestalk and the brain (Table 3).

### Neuropeptide Y (NPY)

**Figure 15:**
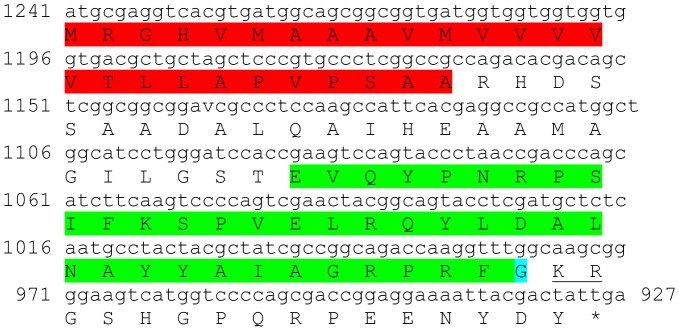
Neuropeptide Y (NPY) precursor predicted complete ORF. A complete NPY precursor (derived from Unigene30121_All) starting with a signal peptide (red) followed by a Pancreatic hormones/neuropeptide F/peptide YY family domain (green) with an amidated glycine (light blue). Asterisk indicates the stop codon.

One transcript was identified to putatively encode a complete NPY precursor with 104 aa (Table 1 and Fig. 15). The sequence starts with a 26 aa signal peptide followed by a 36 aa pancreatic hormone/neuropeptide F/peptide YY family domain, which showed 57% identity with an NPY from a mollusk (Table 2). Neuropeptide Y showed significantly higher expression in the eyestalk compared to the brain (Table 3).

### Neuroparsin

**Figure 16:**
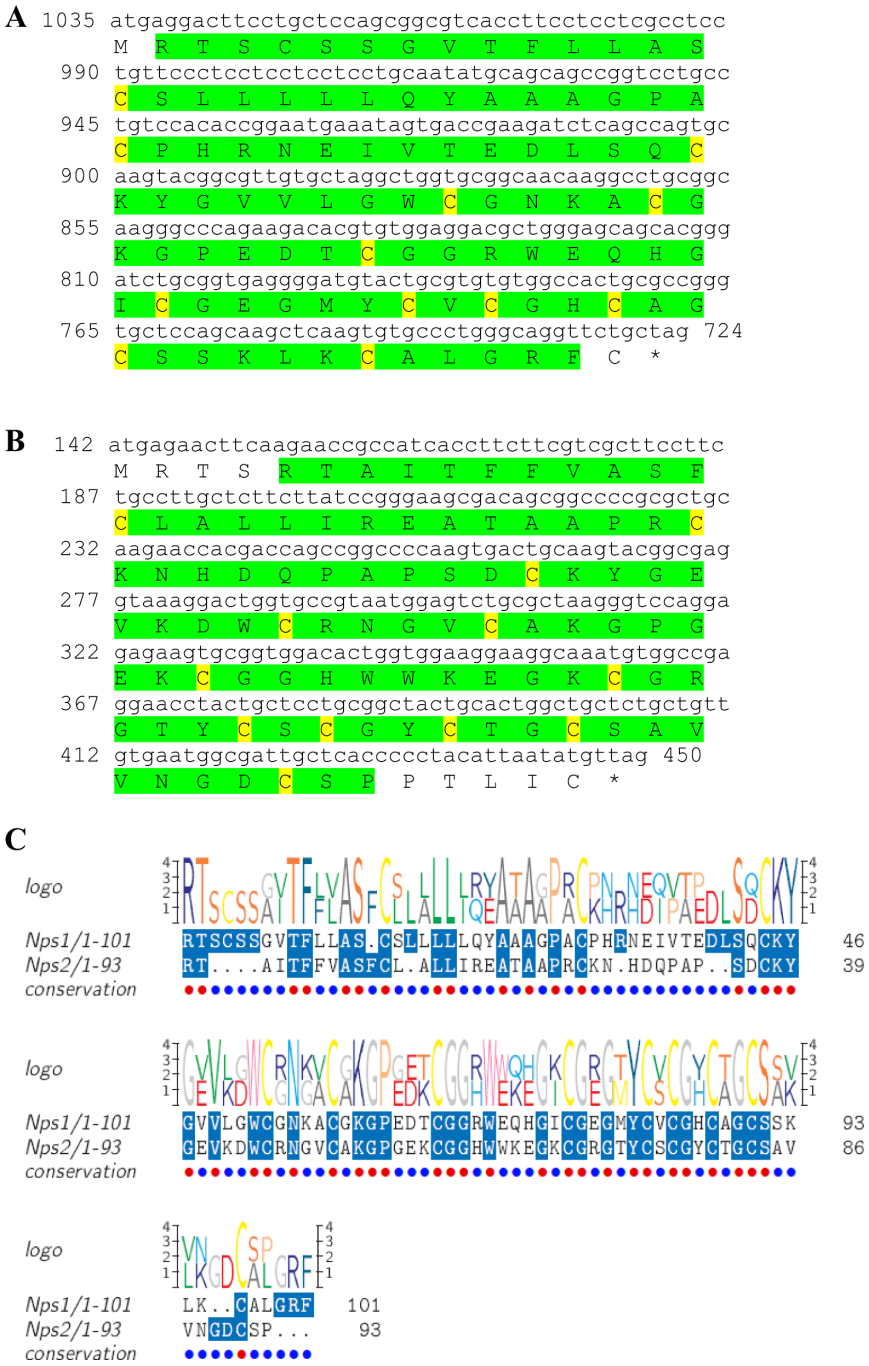
Neuroparsin precursor predicted ORFs and conserved peptide. **A, B**) Complete neuroparsin precursor predicted ORFs (derived from CL2744.Contig6_All and Unigene5705_All) each with a neuroparsin domain (green) with 12 conserved cysteine residues (yellow). Asterisk indicates the stop codon. **C**) Amino acid alignment between the neuroparsin domains.

Two transcripts were identified to putatively encode complete neuroparsin peptide precursors with 103–102 aa (Table 1 and Fig. 16A,B). Both sequences contain a 93–101 aa neuroparsin domain with very low similarity (44% identity), although all 12 cysteine residues, predicted to form 6 disulfide bridges are aligned (Fig. 16C). Although the similarity between the two isoforms was rather low, both showed similarity to the same neuroparsin of a spiny lobster (97% and 48%; Table 2). The first neuroparsin encoding transcript had higher expression compared with the second transcript. In both cases the expression was not significantly different between tissues, due to high variation between males and females (Table 3).

### Orcokinin

**Figure 17:**
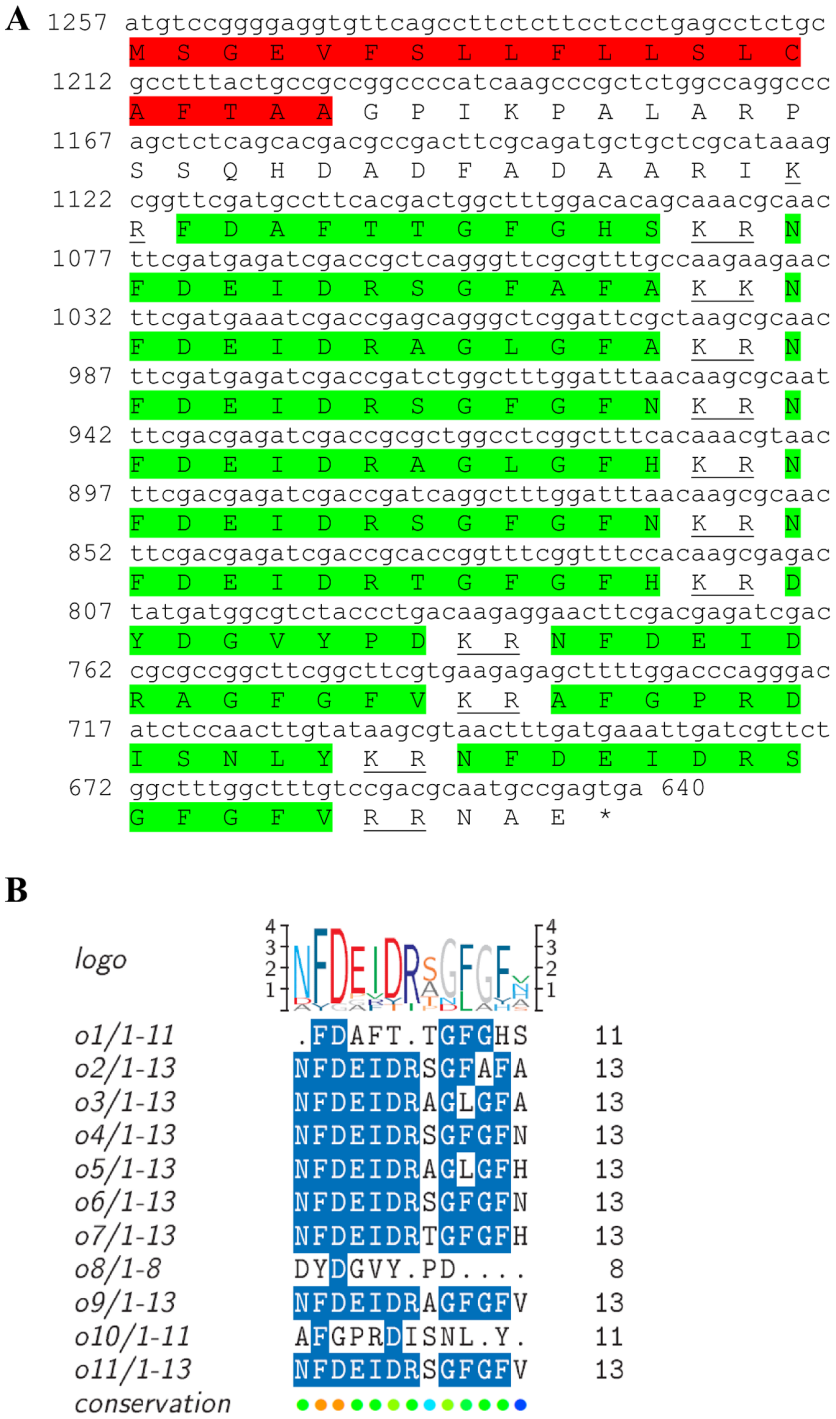
Orcokinin precursor predicted complete ORF and conserved motif. **A**) A complete ocrcokinin precursor predicted ORF (derived from Unigene692_All) with signal peptide (red) and 11 predicted orcokinin peptides (green), separated by carboxyl-peptidase cleavage sites (underlined) Asterisk indicates the stop codon. **B**) Orcokinin peptides conservation: 11 predicted neuropeptides of 8–13 aa in length with **NFDEIRDR**X**GFGF**X conserved.

One transcript was identified to putatively encode a complete orcokinin peptide precursor with 205 aa (Table 1 and Fig. 17), starting with a signal peptide of 20 aa, followed by 11 putative neuropeptides, separated by dibasic proteinase cleavage sites (Fig. 17A). The predicted neuropeptides are 8–13 aa in length with **NFDEIRDR**X**GFGF**X as the most conserved motif (Fig. 17B). All 11 neuropeptides had high homology (5 identical) with orcokinin of either the clawed lobster *Homarus americanus* or the red swamp crayfish *Procambarus clarkii* (Table 2). Orcokinin showed higher expression in the male brain compared with the female brain, with similar expression in the eyestalk and the brain (Table 3).

### Pigment dispersing hormone (PDH)

**Figure 18:**
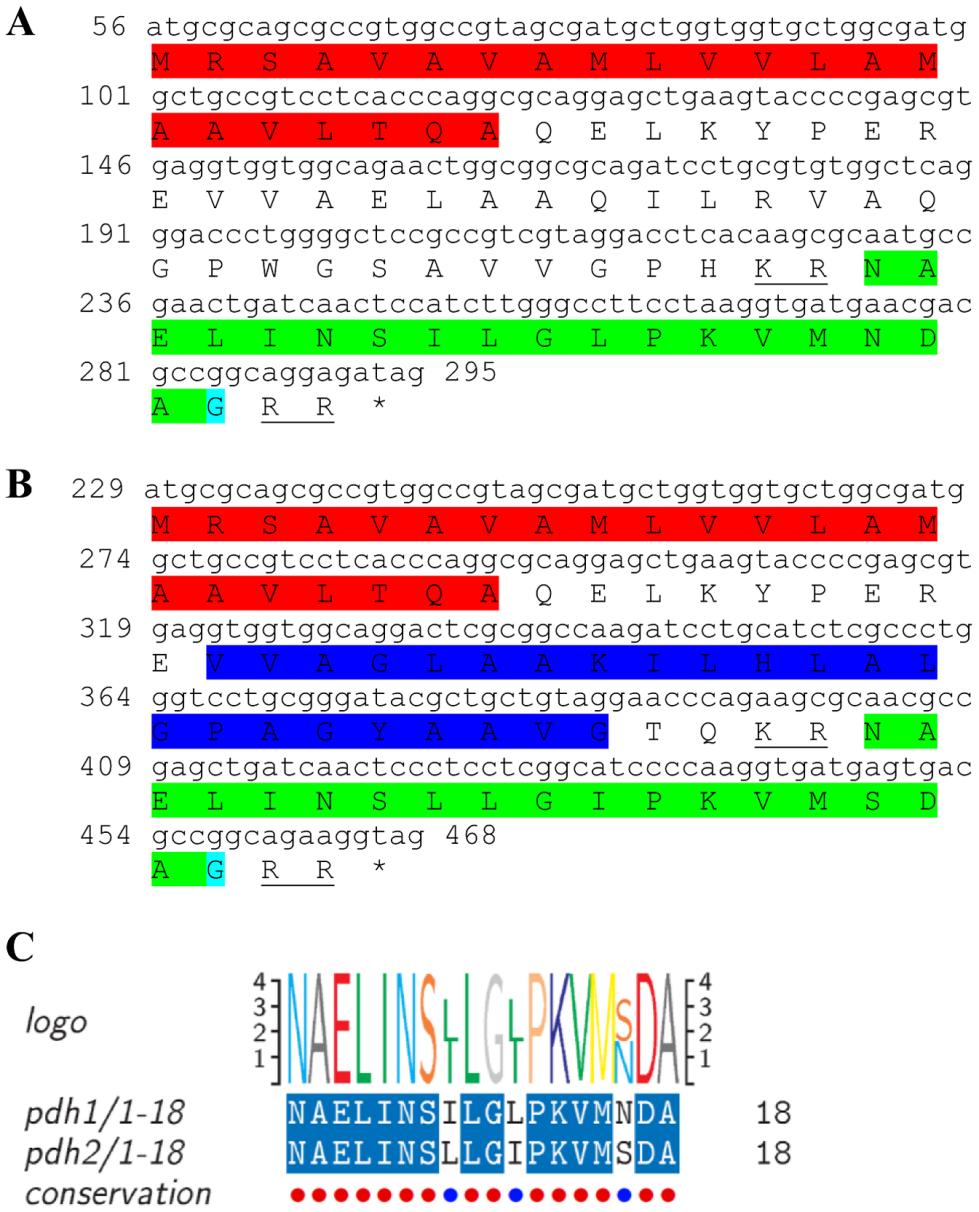
PDH precursor predicted complete ORFs and conserved motif. **A, B**) Two complete PDH precursor predicted ORFs (derived from CL7594.Contig2_All and CL7594.Contig3) each starting with an identical signal peptide (red), a transmembrane region in one isoform (dark blue) and a predicted PDH peptide (green), preceded by a carboxyl-peptidase cleavage site (underlined) in each predicted isoform with an amidated glycine (light blue). Asterisk indicates the stop codon. **C**) PDH peptides conservation 15/18 aa are identical with the other 3 similar in characteristics.

Two transcripts were identified to putatively encode complete, highly similar isoforms of PDH precursors (Table 1 and Fig. 18) with 79 aa, both starting with an identical signal peptide of 22 aa, followed by a 23 aa transmembrane region in only one isoform, followed by a carboxy-peptidase cleavage site prior to an 18 aa PDH domain in both isoforms (Fig. 18A, B). Of the18 aa’s, 15 are identical and the other 3 are similar (Fig. 18C). Both neuropeptides had high homology with previously identified PDH of decapod crustaceans (Table 2). Both of the PDH encoding transcripts showed significantly higher expression in the eyestalk compared with the brain and a higher level in the male brain compared with the female brain.

### Prohormone-3

**Figure 19:**
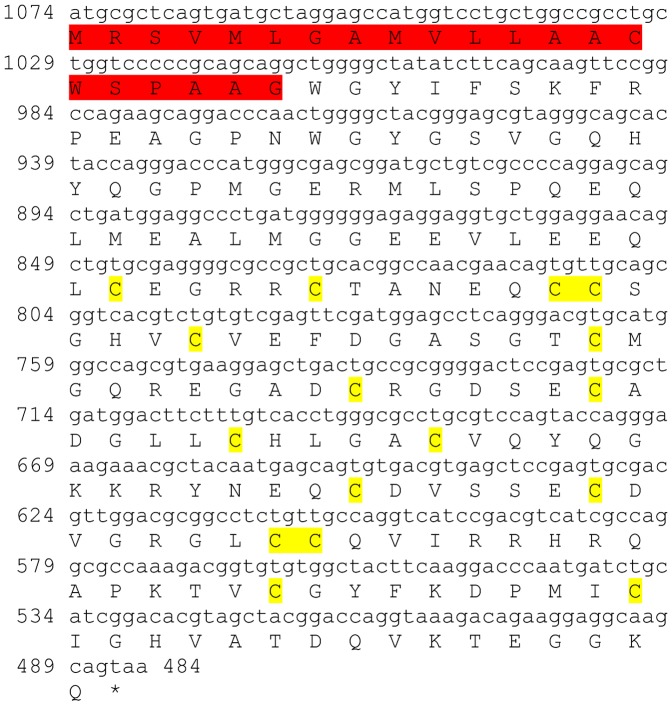
Prohormone-3 precursor predicted complete ORF. A complete prohormone-3 peptide precursor (derived from CL1958.Contig1_All) with a signal peptide (red) and 12 cysteine residues (yellow). Asterisk indicates the stop codon.

One transcript was identified to putatively encode a complete prohormone-3 peptide precursor with 196 aa (Table 1 and Fig. 19). The sequence starts with a 21 aa signal peptide and contains 12 cysteine residues (Fig. 19), all conserved with other insect prohormone-3 sequences, with up to 43% identity in sequence (Table 2). Prohormone-3 encoding transcript showed higher expression in the eyestalk compared to the brain, with higher expression in the male brain compared with the female brain (Table 3).

### Prohormone-4

**Figure 20:**
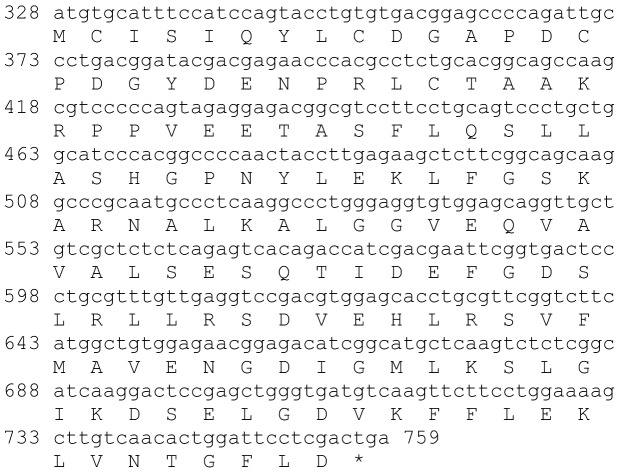
Prohormone-4 precursor predicted partial ORF. A partial prohormone-4 peptide precursor (derived from Unigene19311_All). Asterisk indicates the stop codon.

One transcript was identified to putatively encode a partial C-terminus of prohormone-4 peptide precursor with 143 aa (Table 1 and Fig. 20). The highest homology to an insect species was 89% (Table 2). Prohormone-4 encoding transcript showed higher expression in the brain compared to the eyestalk, with higher expression in the male compared with the female, in both eyestalk and brain (Table 3).

### Red pigment concentrating hormone (RPCH)

**Figure 21:**
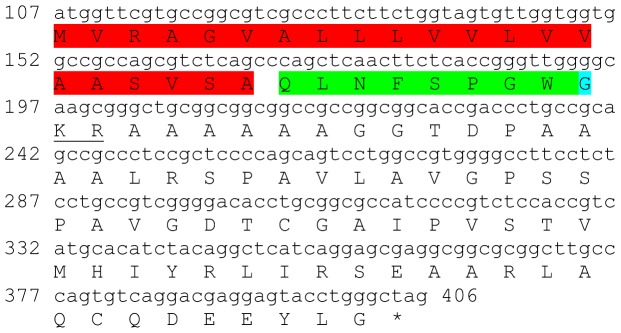
RPCH precursor predicted complete ORF. A complete RPCH peptide precursor (derived from Unigene2547_All) starting with a signal peptide (red) followed by a RPCH domain (green) with an amidated glycine (blue). Asterisk indicates the stop codon.

One transcript was identified to putatively encode a complete RPCH peptide precursor with 99 aa (Table 1 and Fig. 21). The sequence starts with a 21 aa signal peptide followed by the 8-residue RPCH peptide (with 100% identity to peptides of other RPCHs) and RPCH-associated peptide C-terminal domain (Fig. 21). The overall prohormoe shared 63% identity with the blue swimmer crab *Callinectes sapidus* RPCH (Table 2). Red pigment concentrating hormone encoding transcript showed higher expression in the eyestalk compared to the brain (Table 3).

### Sulfakinin

**Figure 22:**
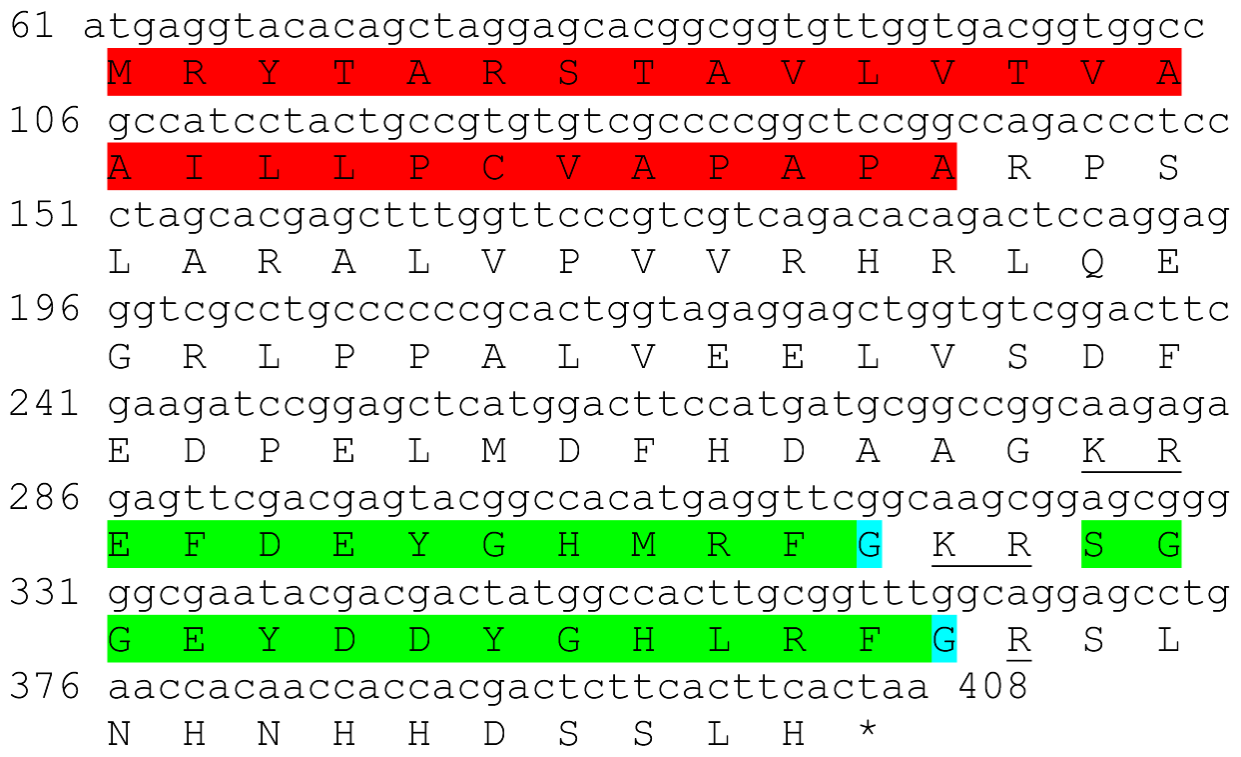
Sulfakinin precursor predicted complete ORF. A complete sulfakinin peptide precursor (derived from Unigene25008_All) starting with a signal peptide (red) followed by two sulfakinin putative peptides (green) with an amidated glycine (blue), separated by putative carboxy-peptidase cleavage sites (underlined). Asterisk indicates the stop codon.

One transcript was identified to putatively encode a complete sulfakinin peptide precursor with 115 aa (Table 1 and Fig. 22). The sequence starts with a 27 aa signal peptide followed by two sulfakinin putative peptides of 10 aa and 13 aa, separated by carboxy-peptidase cleavage sites (Fig. 22). The two peptides had high homology with sulfakinin of *H. americanus* (Table 2). Sulfakinin encoding transcript showed higher expression in males compared to females both in the brain and the eyestalk (Table 3).

### Tachykinin

**Figure 23:**
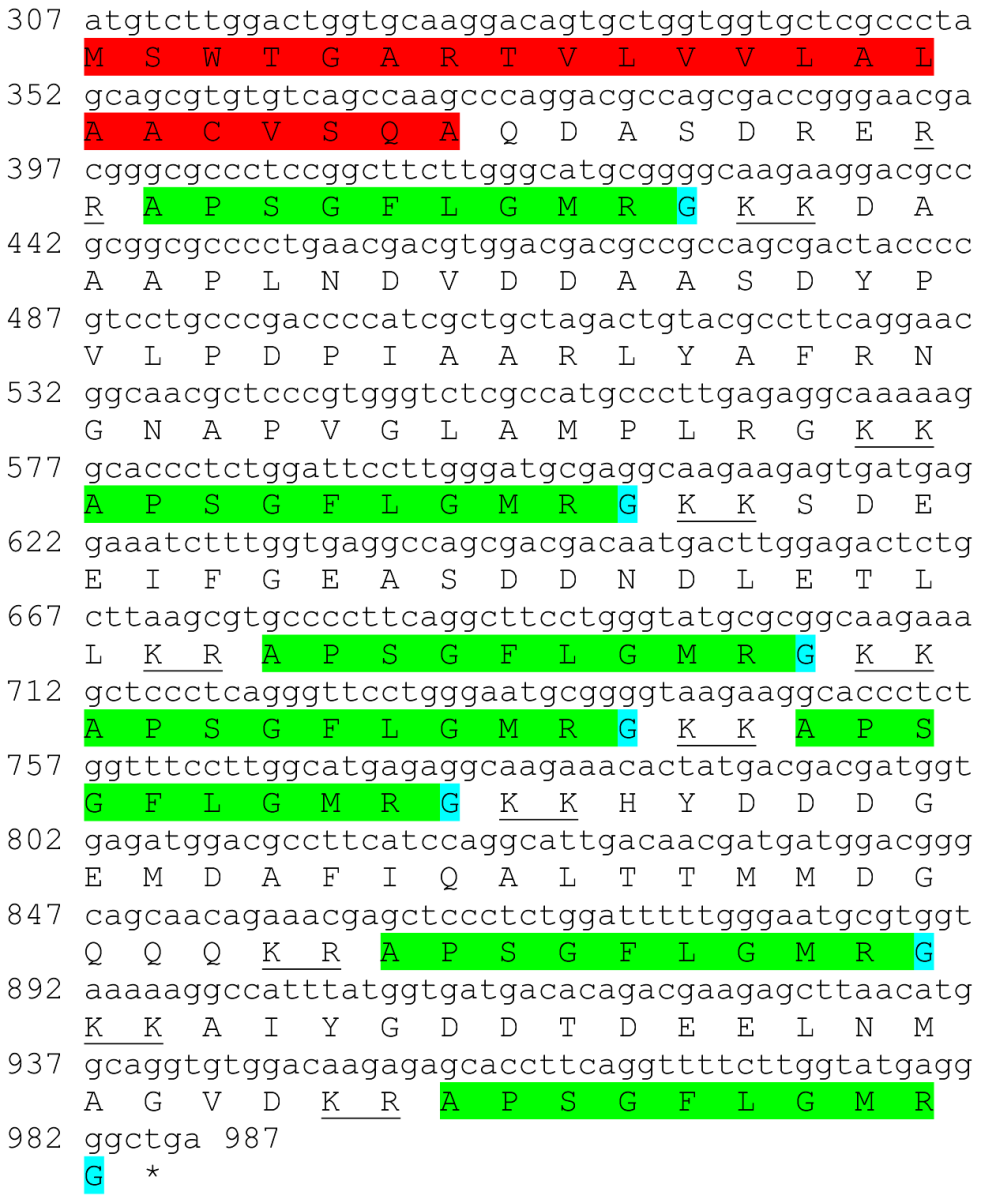
Tachykinin precursor predicted complete ORF. A complete tachykinin peptide precursor (derived from CL7656.Contig2_All) starting with a signal peptide (red) followed by seven identical tachykinin putative peptides (green) with an amidated glycine (blue), separated by putative carboxy-peptidase cleavage sites (underlined). Asterisk indicates the stop codon.

One transcript was identified to putatively encode a complete tachykinin peptide precursor with 226 aa (Table 1 and Fig. 23). The sequence starts with a 22 aa signal peptide followed by seven identical tachykinin putative peptides of 9 aa each (APSGFLGMRamide), separated by carboxy-peptidase cleavage sites (Fig. 23). This peptide was found to be identical to the tachykinin found in *P. clarkii* (Table 2). Tachykinin encoding transcript showed significantly higher expression in the brain compared with the eyestalk (Table 3).

## Discussion

This study has elucidated the putative neuropeptidome of the previously uncharacterized Eastern rock lobster *S. verreauxi*. Overall 37 partial and complete transcripts were identified which putatively encode 21 peptide families/sub-families (Table 1). These included three partial **allatostatin type A** transcripts, where one is presumed to represent the N-terminus (Fig. 1A), the other is presumed to represent the middle region (Fig. 1B) and the third is presumed to represent the C-terminus (Fig. 1C). It is conceivable that these three transcripts are part of a one, larger transcript which includes all three, as in most studied arthropod species only one type A allatostatin gene was identified [22], except for blowflies [23]. Overall there are 22 mature peptides of 8 aa predicted to arise from the above three transcripts, each containing the highly conserved YXFGLamide motif (Fig. 1D), found in all arthropods type A allatostatins [22]. Two partial peptides were identified as the putative N-terminus and C-terminus of **type B allatostatin** precursors (Fig. 2A and B, respectively). The level of conservation between the 13 putative mature peptides encoded by these transcripts was much lower compared with the conservation between the predicted type A allatostatins and six are novel (Fig. 2C). Two transcripts were identified to encode complete **type C allatostatin** precursors with very low conservation between the two predicted mature peptides which include the signature cysteine residues of the type C allatostatins (Fig. 3A, B, C). The latter sequence whose best BLAST hit was the predicted prohormone-1 of the honey bee (Table 1) includes the predicted mature peptide which is broadly conserved among crustaceans SYWKQCAFNAVSCFamide [24]. Most of the mature peptides had very high homology with other arthropods, primarily other decapod crustacean species. Most prominent was the conservation of type A allatostatine-derived peptides with those of the spiny lobster *P. interruptus* and the broadly conserved peptide in prohormone-1 (Table 2).

One complete **bursicon alpha subunit** predicted sequence was identified, containing a signal peptide and a predicted C-terminal cysteine knot-like domain (Table 1, Fig. 4) with 11 cysteine residues well conserved with other crustacean and insect species, 10 of which are hypothesized to form five disulfide bridges [25]. Another transcript is hypothesized to be the N- terminus part of a **corazonin** precursor, comprising a signal peptide, followed by the 11 aa conserved peptide which is the signature of corazonin (QTFQYSRGWTNamide) [26], followed by a carboxy-peptidase cleavage site (Table 1 and Fig. 5). Another sequence is predicted to encode the crustacean cardioactive peptide precursor (**CCAP**), with 139 aa and high similarity to other crustacean sequences (Table 1&2, Fig. 6).

Five sequences were identified to encode four predicted complete and near complete **type B CHH** precursors (Crustacean hyperglycemic hormones) and another unspecified CHH precursor. The putative peptides were identified to be specific to the eyestalk as expected from CHHs and included a signal peptide (in 4 out of 5 sequences) and a conserved CHH domain (Table 1, Fig. 7). Although the occurrence of splice variance-derived isoforms of CHH is well documented [27], we currently cannot rule out that the high similarity between the 5 sequences identified (up to 89% identity) is due, at least in part, to sequencing/assembly errors rather than actual isoforms. Three sequences were identified to putatively encode complete isoforms of Molt/Gonad-inhibiting hormone (**MIH/GIH**). All predicted isoforms included a signal peptide followed by a conserved MIH/GIH domain with intermediate similarity (up to 54% identity; Table 1, Fig. 8), suggesting these are more reliably representing isoforms, compared with the predicted CHHs. The homology of CHHs and MIHs with others identified in decapod crustaceans was in some cases higher than the homology between the isoforms themselves (Table 2), consistent with these genes being diverged for a long time. Most CHH and MIH isoforms were found to be expressed predominantly in the eyestalk with three of the CHH isoforms and one MIH isoform that are most abundantly expressed (Table 3). In most isoforms higher expression was found in females, suggesting that the females sampled were more advanced in the molt cycle. Repeating the neuropeptidome analysis with more samples of males and females of distinct molt stages will enable better distinction between neuropeptides whose expression change with relation to molt cycle and neuropeptides whose expression change between genders. Another sequence which was found to express specifically in the eyestalk was predicted to encode a complete Crustacean female sex hormone precursor (**CFSH**; Table 1, Fig. 9). CFSH was recently identified in two brachyuran crabs and was found to be specifically expressed in the female eyestalk. CFSH knock-down was shown to inhibit the appearance of the female reproductive characteristics which accompany the terminal molt in these species (GenBank Accession # ADO00266). Interestingly, the putative CFSH in *S. verreauxi*, identified in this study, was found to be specific to the eyestalk although it is present also in male eyestalks with the same level of expression as in females.

One transcript was predicted to encode a complete calcitonin-like diuretic hormone (**DH**), with high similarity to the one identified in the American lobster *H. americanus* [28] (Table 1, Fig. 10). Two transcripts were predicted to encode two complete **eclosion hormone** precursor isoforms (with 47% identity) each starting with a signal peptide and containing 6 conserved cysteine residues within their eclosion hormone domain (Table 1, Fig. 11). Two transcripts were predicted to encode **follistatin**-like peptides. Although not considered as neuropeptides, these were included here as it might be of interest to further pursue their precise functionality in crustaceans. The N-termini of both predicted isoforms include identical signal peptides, followed by identical follistatin domains, followed by a kazal-type serine protease inhibitor domain whose N- terminus is identical and the C- terminus was different (Table 1, Fig. 12). One isoform includes a predicted transmembrane region and is a complete ORF (Fig. 12A), while the other is longer, without a predicted transmembrane region and a partial ORF (Fig. 12B). One transcript was identified to encode a complete **myostatin** precursor with the exact same sequence of that identified in the penaeid shrimp *P. monodon* (Table 1, Fig. 13). Although also not considered a neuropeptide, like follistatin, its function in regulating muscle development in crustaceans is an interesting aspect to pursue and is thus included here. Recently, an opposite role was assigned to myostatin in *P. monodon* compared with vertebrates [29]. Based on the identical sequence identified in this study, the Eastern rock lobster might serve a good candidate species to revisit this hypothesis. A complete **myosupressin** precursor was predicted with a signal peptide and high similarity with *H. americanus* myosupressin (Table 1, Fig. 14).

One complete predicted neuropeptide Y (**NPY**) precursor was identified with a conserved active peptide sequence (Table 1, Fig. 15) and two predicted **neuroparsin** complete peptide precursors were identified with 12 conserved cysteine residues in each, but with rather intermediate similarity between them (Table 1, Fig. 16). Another predicted neuropeptide, **orcokinin** was identified that included a highly conserved motif of NFDEIRDRXGFGFX within its 11 predicted mature peptides (Table 1, Fig. 17). Two isoforms of the pigment dispersing hormone (**PDH**) precursor were identified with intermediate similarity overall. The predicted mature peptide shows high similarity between the two sequences (15/18 aa identical). Two sequences were predicted to encode complete **prohormone-3** and **prohormone-4** precursors (Table 1, Fig. 19, 20). Both have been characterized solely in insects, apart from one prohormone-4 like peptide identified in the copepod *Acartia pacifica* (GenBank accession number AGN29584), hence this is the first report of the two hormones in decapods.

A predicted red pigment concentrating hormone (**RPCH**) precursor was identified with a signal peptide and RPCH domain (Table 1, Fig. 21). Another sequence is predicted to encode a complete **sulfakinin** precursor with a signal peptide and two mature peptides separated by peptidase cleavage sites (Table 1, Fig. 22). Finally, one sequence was identified to putatively encode a complete **tachykinin** precursor with a signal peptide followed by seven identical tachykinin peptides, separated by peptidase cleavage sites (Table 1, Fig. 23). The tachykinin putative sequence had high similarity to the one identified in the spiny lobster *P. interruptus*.

Diuretic hormone, eclosion hormone, orcokinin, pigment dispersing hormone, prohormone-3, prohormone-4 and sulfakinin all show higher expression levels in males, while CHH and MIH show higher expression levels in females (Table 3). Further analysis in precise molt stages is required to validate if these neuropeptides have only a role in molt regulation or are also modulating gender-derived differences. This study have laid the foundations that will enable us to pursue this biological question.

## Conclusions

This study describes a comprehensive transcriptome of the central nervous system of *S. verreauxi* whose mining led to the identification of its putative neuropeptidome. Most of the identified neuropeptides had high similarity with previously identified neuropeptides, primarily those of other closely-related decapod crustaceans. Approximately 21 families and sub-families were covered, including neurohormones previously identified in other crustacean species as well as two that were previously reported primarily in insects and this is the first report of their identification in decapod crustaceans (prohomone-3 and 4). Mapping and quantification gives insights into the dynamics of neuropeptides expression during the molt cycle and with regards to gender.

## Materials and Methods

### Animals

*Sagmariasus verreauxi* individuals were maintained at Institute for Marine and Antarctic Studies under previously described parameters [30]. Prior to dissections, animals were anesthetized on ice for at least 20 min.

### Sample Preparation and Sequencing

Total RNA from eyestalks and brains of two mature *S. verreauxi* males and two mature females were isolated separately with the Trizol Reagent (Invitrogen), according to the manufacturer's instructions, followed by next generation sequencing by BGI (HongKong Co. Ltd) as per manufacturer’s protocol (Illumina, San Diego, CA). Briefly, poly (A) mRNA was isolated using oligo (dT) beads and the addition of fragmentation buffer for shearing mRNA into short fragments (200–700 nt) prevented priming bias during the synthesis of cDNA using random hexamer-primers. The short fragments were further purified using QiaQuick PCR extraction kit and resolved with EB buffer for ligation with Illumina Paired-end adapters. This was followed by size selection (∼200 bp), PCR amplification and Illumina sequencing using an Illumina Genome Analyzer (HighSeq 2000, Illumina, San Diego, CA), performing 90 bp–paired end sequencing. The sequence reads were stored as FASTQ files. Overall, at least 4 Gb of cleaned data (at least 45 million reads) was generated for each of the four samples sequenced, which included pooled eyes of two males and two females, pooled brains of two males and two females.

### Bioinformatics analyses

Cleaning of low quality reads, assembly and annotation were done by BGI, using unpublished algorithms (BGI, HongKong Co. Ltd), Trinity [31] and Blast2GO [32], respectively. We validated that the reads obtained by BGI are clean using FASTQ/A Trimmer (http://hannonlab.cshl.edu/fastx_toolkit/index.html), which gave an output of over 99.99% of the reads untrimmed. The list of annotated sequences was scanned for key words, including names and abbreviations of previously known neurohormones as well as general key words such as 'hormone'. Multiple sequence alignment of the predicted neuropeptide sequences was performed with ClustalW [33], followed by a Neighbor Joining Phylogram (for the CHH sequences) generated via MEGA 5.0 [34] with 1000 bootstrap trials. The multiple sequence alignment file was then exported to TexShade [35] for highlighting the conserved sequence motifs. Signal peptide was predicted using SignalP 4.1 server [36]. Domain prediction was done either via SMART [37] or by comparison with references of other crustacean neuropeptide sequences. The re-validated clean FASTQ files were re-assembled using default parameters in CLC Genomics Workbench v4 (CLC Bio) and validated the assembled transcripts corresponding the neuropeptides using BLAST. Digital Gene Expression was computed using CLC Genomics Workbench v4 (CLC Bio), with default parameters with the exception of 0.9 similarity fraction instead of 0.8. Resulting BAM files were deposited in the sequence read archive (http://www.ncbi.nlm.nih.gov/sra) as biosample SAMN02419461. BAM files were then uploaded onto Partek Genomics Suite (Partek GS) where quantification was performed, yielding reads per kilobase per million reads (RPKMs). The quantified data was analyzed using ANOVA, performed in Partek GS, with contrast between values in eye and brain for each neuropeptide. The threshold for statistical significance was set to p<0.05. Since there was only one male and one female sample for each tissue, no statistical analysis was applicable to compare males and females.
